# Genomic Variation among Strains of *Crithidia bombi* and *C. expoeki*

**DOI:** 10.1128/mSphere.00482-19

**Published:** 2019-09-11

**Authors:** Evgeny Gerasimov, Niklaus Zemp, Regula Schmid-Hempel, Paul Schmid-Hempel, Vyacheslav Yurchenko

**Affiliations:** a Martsinovsky Institute of Medical Parasitology, Tropical and Vector Borne Diseases, Sechenov University, Moscow, Russia; b Faculty of Biology, M. V. Lomonosov Moscow State University, Moscow, Russia; c Genetic Diversity Centre, ETH Zürich, Zürich, Switzerland; d Institute of Integrative Biology, ETH Zürich, Zürich, Switzerland; e Life Science Research Centre, Faculty of Science, University of Ostrava, Ostrava, Czech Republic; University at Buffalo

**Keywords:** *Crithidia*, *Trypanosomatidae*, genomics

## Abstract

In this study, we sequenced and analyzed the genomes of 40 strains, in addition to the already-reported two type strains, of two *Crithidia* species infecting bumblebees in Alaska and Central Europe and demonstrated that different strains of Crithidia bombi and C. expoeki vary considerably in terms of single nucleotide polymorphisms and gene copy number. Based on the genomic structure, phylogenetic analyses, and the pattern of copy number variation, we confirmed the status of *C. expoeki* as a separate species. The Alaskan populations appear to be clearly separated from those of Central Europe. This pattern fits a scenario of rapid host-parasite coevolution, where the selective advantage of a given parasite strain is only temporary. This study provides helpful insights into possible scenarios of selection and diversification of trypanosomatid parasites.

**IMPORTANCE** A group of trypanosomatid flagellates includes several well-studied medically and economically important parasites of vertebrates and plants. Nevertheless, the vast majority of trypanosomatids infect only insects (mostly flies and true bugs) and, because of that, has attracted little research attention in the past. Of several hundred trypanosomatid species, only four can infect bees (honeybees and bumblebees). Because of such scarcity, these parasites are severely understudied. We analyzed whole-genome information for a total of 42 representatives of bee-infecting trypanosomatids collected in Central Europe and Alaska from a population genetics point of view. Our data shed light on the evolution, selection, and diversification in this important group of trypanosomatid parasites.

## Abstract (precis)

A group of trypanosomatid flagellates includes several well-studied medically and economically important parasites of vertebrates and plants. Nevertheless, the vast majority of trypanosomatids infect only insects (mostly flies and true bugs) and, because of that, has attracted little research attention in the past. Of several hundred trypanosomatid species, only four can infect bees (honeybees and bumblebees). Because of such scarcity, these parasites are severely understudied. We analyzed whole-genome information for a total of 42 representatives of bee-infecting trypanosomatids collected in Central Europe and Alaska from a population genetics point of view. Our data shed light on the evolution, selection, and diversification in this important group of trypanosomatid parasites.

## INTRODUCTION

Monoxenous trypanosomatid flagellates (with one host, usually an insect, in their life cycle) have, for a long time, been considered dull and not-so-interesting cousins of the economically or medically important dixenous kin that have two hosts in their life cycle, an insect and a vertebrate or a plant (1, 2). This view has been challenged recently when insect parasites were found to be coinfecting vertebrates (3–5) or as their ancestral role in the evolution of dixeny became recognized (6, 7).

The monoxenous trypanosomatid parasites of bees are rather unique in many regards. This group of *Leishmaniinae* spp. (8) (which includes Crithidia bombi, C. expoeki, C. mellificae, and Lotmaria passim) are the only trypanosomatids able to infect hymenopteran insects. *Crithidia mellificae* and *Lotmaria passim* infect honeybees (*Apis* spp.) (9, 10), whereas *C. bombi* and *C. expoeki* are common parasites of bumblebees (*Bombus* spp.) (11, 12). All of these parasites have a worldwide distribution.

Bumblebees are social insects, and individual infections are acquired outside the nest by the foraging workers when visiting flowers that had been previously visited by an infected bee or by contact with contaminated food or nest material inside the colony (13). In general, the infection has mild effects on the workers, but it can have severe consequences for the founding queens in spring, since they become unable to found a colony (14). There are concerns that the presence of this infectious pathogen contributes to the decline in the populations of wild bumblebees in the United States (15), South America (16), or the United Kingdom (17).

A particular hallmark of this biological system is the strong effect of the host and parasite genotypic background on a success of infection. Bumblebees colonies vary considerably in their susceptibility to infection and, vice versa, different genotypes (“strains”) of *Crithidia* spp. vary in the range of different host colonies they are able to infect (18). This effect can be explained by variation in the genotypes (19, 20) and the corresponding gene expression patterns (21, 22). Among other factors, genotypic variation in parasite populations is determined by recombination during genetic exchange among coinfecting strains (23, 24) and maintained by selection during passage within colonies (25–27). As a result, natural bumblebee populations harbor a wide range of parasite’s genotypes, even to the extent that each newly typed infection can be another unique genotype (28, 29).

The genomes of *Crithidia bombi* and *C. expoeki* were sequenced recently and demonstrated a considerable synteny in their organization (30). Several dozen orthologous gene groups, defined across a set of trypanosomatid species, revealed signatures of positive selection in the branch leading to *Crithidia*. Genes, putatively involved in the host-parasite interaction, were shown to coevolve. Remarkably, in *C. bombi* the biosynthesis of cell surface components, which are important for host-parasite interactions, deviates from that in most other eukaryotes, indicating the effects of rapid host-parasite coevolution (30).

Although previous studies, based on microsatellite polymorphism, have shown that genotypic variation is a major element of the natural interactions of these parasites with their bumblebee hosts, the extent of genomic differences among different strains of *Crithidia* has not yet been investigated in detail. Here, we compared the full genome sequences of 40 strains of *C. bombi* and *C. expoeki* that were collected at different sites, plus the genomes of the two type strains. We show that the analyzed strains form clusters with shallowly separated branches, show variation in gene copy numbers, and possess genes under positive selection.

## RESULTS

### Strain collection and sequencing.

Previously, we have sequenced two strains of two *Crithidia* spp. using a combination of PacBio, Illumina, and Roche 454 technologies, and we assembled two high-quality reference genomes (30). We will further refer to BJ08_175 as a type strain for *C. expoeki* and to 08_076 as a type strain for *C. bombi*.

We sequenced 41 genomes using the Illumina platform with paired-end sequencing protocol and high coverage. Of these, one genome (BJ08_175a) was a repetition of the genome of the same strain, leaving 42 different strains (40 + 2 type strains) available (see Table S1 in the supplemental material). These strains were collected mainly in Switzerland (plus two samples collected on the Mediterranean islands of Corsica and Sardinia that were included in the “Switzerland strain” group based on their position in the phylogenetic tree, as described below) and in Alaska. The technical parameters of processing the data for newly sequenced strains are summarized in Table S2. The average coverage is about 95×, which is adequate for both single nucleotide polymorphism (SNP) calling and copy number variation (CNV) analyses (except for strain BJ08_068, with an average coverage of 51×, for which the CNV analysis was not performed).

10.1128/mSphere.00482-19.1 TABLE S1 List of clones used in this study. Code numbers used in the overall long-term projects, *Crithidia* type (*cytB* haplotype), host specificity (A stands for *Crithidia bombi*, and B for *C. expoeki*), origin (Switzerland [CH], Alaska, Corsica, or Sardinia) and dates of collection are shown. The type specimen for the genomic sequences of *C. bombi* (08_076) and *C. expoeki* (BJ08_175) are in boldface. Clone shown in italic (B08_175a) is a repeat.^1^Code refers to ID of the clone used in the long-term data bank of the ETH lab. ^2^See reference 11 for details on the types. ^3^Castes are spring queens (Q) or workers (W). ^4^This strain was not used for SNP calling and CNV analysis due to lower coverage. Download Table S1, DOCX file, 0.02 MB. Copyright © 2019 Gerasimov et al. 2019 Gerasimov et al. This content is distributed under the terms of the Creative Commons Attribution 4.0 International license.

10.1128/mSphere.00482-19.2 TABLE S2 Technical information on mapping and SNP calling. Data for 40 strain sequenced in frame of the current work. Two strains (AK08_053 and 08_091) were sequenced as two independent biological replicates. Light sky blue color denotes strains of *Crithidia bombi* (lineage A [11]), moccasin color denotes strains of *C. expoeki* (lineage B [11]). Table shows technical information on read mapping and SNP calling for nuclear and mitochondrial maxicircle genomes. In SNP calling statistics, the ‘SNP, homozygote’ and ‘SNP, heterozygote’ show the numbers of homozygous and heterozygous SNPs (with respect to reference type strain) in current strain (the sum of the values gives total number of SNPs in the strain). ‘SNP, reference’ column gives the number of covered genomic positions that are variable in other strains, but are in reference state in the current strain. The sum of all three ‘SNP’ columns gives the total number of SNPs, detected in all strains pool (165,124 variable genome positions in 27 strains of *Crithidia bombi* and 47,725 positions in 14 *Crithidia expoeki* strains). Columns ‘Transitions’, ‘Transversions’, and ‘Ts/Tv’ classify SNPs and give transition-to-transversion ratio. The ‘SNPs, %’ column shows the proportion of SNPs in current sample from all variable genomic positions (SNP pool). The last two columns show the genome variation measured as SNP density, which is an average number of nucleotides that should be taken from genome to find 1 SNP. Overall variation is shown as histogram plotting (1/SNP density) for each strain. Download Table S2, PDF file, 0.6 MB. Copyright © 2019 Gerasimov et al. 2019 Gerasimov et al. This content is distributed under the terms of the Creative Commons Attribution 4.0 International license.

### Species assignment and read mapping.

**FIG 1:**
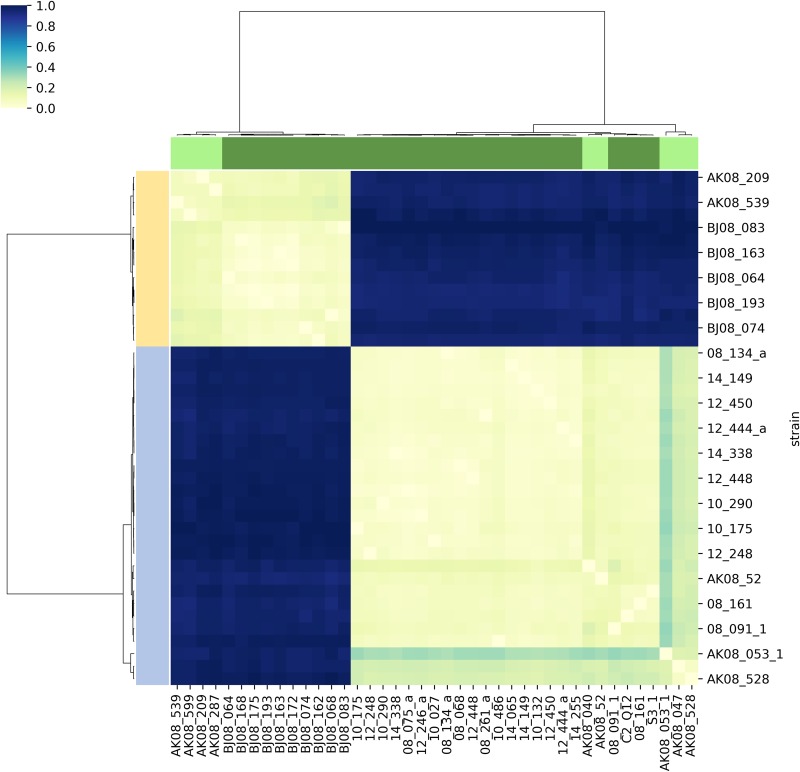
Clustering of sequenced strains based on reference-free SNP calling. Hierarchical clustering of sequenced strains based on distance matrix. Distance matrix obtained from SNP data set generated by DiscoSNP++ software. Distance is scaled to “(0, 1)”. On dendrograms, dark green denotes Switzerland strains, light green denotes Alaskan strains, light blue denotes strains previously genotyped as lineage A, and the moccasin color depicts strains previously genotyped as lineage B (11).

Previously, *18s rRNA*, *ggapdh* (glycosomal glyceraldehyde-3-phosphate dehydrogenase), and mitochondrial *cytB* (cytochrome B) sequences were used to assign the strains to groups (11). These markers defined two distinct lineages A and B, where lineage A corresponded to *Crithidia bombi* and lineage B represented a distinct, newly described species: *C. expoeki*. Here, we first checked whether our previous species assignment remained correct at the whole-genome level. To do that, we called SNPs using raw unassembled reads data for all collected strains with DiscoSNP++ software, which does not rely on a reference genome. This cluster analysis clearly splits the strains into two groups, which correlates with the previous assignments (Fig. 1). Accordingly, the extent of variation within these two species is much lower than between species. The clustering map also revealed that Alaskan strains form separate clades inside the *C. expoeki* clade (previously lineage B [11]) and inside the *C. bombi* clade (lineage A [11]). However, for *C. bombi* the situation was more complex: three Alaskan strains clearly branched out into a separate group, whereas two other Alaskan strains branched in a smaller clade inside the group of European strains.

**FIG 2:**
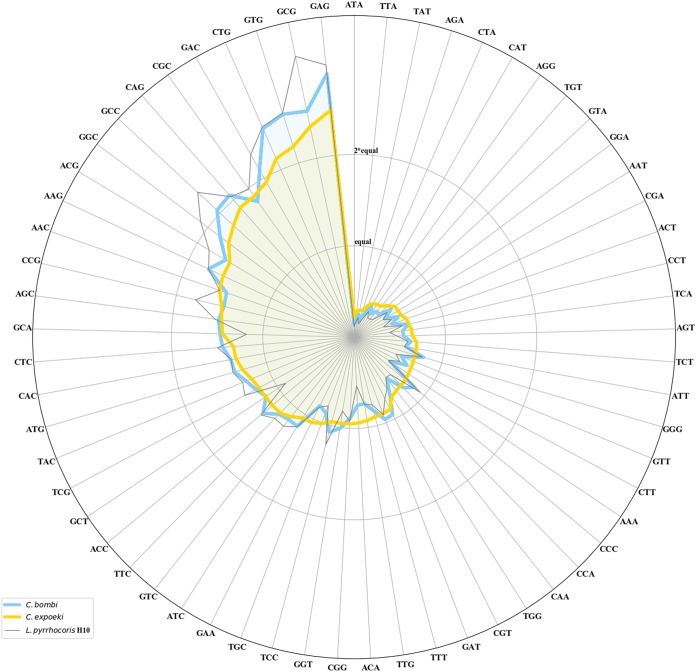
Codon usage plot. Frequencies of codons are plotted on polar coordinate system. Each spike represents a codon. Codons are sorted (counterclockwise) by decrease of frequency used in *C. expoeki* (yellow line). Each strain’s codon usage was plotted individually to show that there is no difference in codon usage between strains. To put the codon usage in context, the usage in *L. pyrrhocoris*, a species from the closest sister group, is plotted in gray.

The codon usage pattern of *C. bombi* clearly differs from that of *C. expoeki* (Fig. 2). The extent of this diversity is comparable to the difference documented between any of the two *Crithidia* spp. and their closest relative, Leptomonas pyrrhocoris. Moreover, the genomic variation between strains does not influence the codon usage patterns, even within the Alaskan strains the usage remains the same. This supports an idea that codon usage is a species-specific trait.

The genomes of the two type strains were used as references for mapping the reads and for the further reference-based SNP calling. Reads for 27 *C. bombi* strains (from a total of 29 samples; one strain was sequenced in two biological replicates, and one strain had insufficient coverage [see Table S1]) and 14 *C. expoeki* strains were taken as independent samples and mapped on their respective genome references. The overall statistics are summarized in Table S2.

### Variant calling and divergence between strains.

Variant calling revealed a total 165,124 variable genome positions in 27 strains of *Crithidia bombi* and 47,725 positions in 14 *C. expoeki* strains. The SNP densities (measured in numbers of nucleotides per SNP and indicating the average distance in nucleotides between SNPs) for *C. bombi* in Switzerland and in two Alaskan strains (AK08_040 and AK08_52) strains were 1,835 and about 1,300 nucleotides (nt), respectively. Of note, three remaining Alaskan strains have much higher SNP densities of approximately 400 nt. The transition/transversion ratio was also higher for the latter three strains (2.74 compared to the average of 2.47 for *C. bombi*; Table S2).

At the same time, *C. bombi* strains demonstrated higher variation than *C. expoeki*. The average SNP distance between any two Switzerland strains is 15,100 SNPs for the former, whereas the SNP density is much lower (1 SNP per 5,500 nt) for the Switzerland strains of *C. expoeki*, with an average distance between two strains of ∼6,000 SNPs. Similar to the situation in *C. bombi*, Alaskan strains of *C. expoeki* have ∼4 times higher SNP density than the Switzerland strains.

For *C. expoeki*, 2,405 genes contained only Alaska-specific SNPs, 652 genes contained only Switzerland-specific SNPs, and 1,836 genes contained both Alaska-specific and Switzerland-specific SNPs. For *C. bombi* these values were 1,243, 799, and 5,084, respectively.

**FIG 3:**
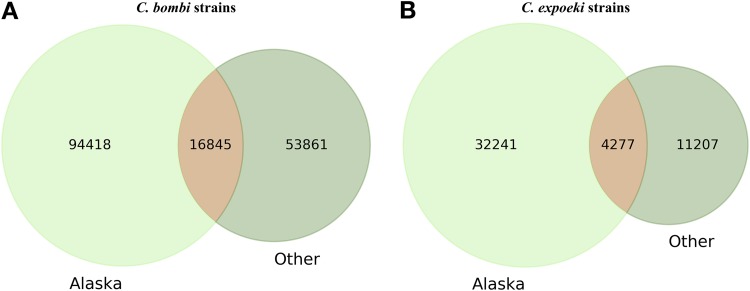
SNP distribution among populations. Venn diagrams for *Crithidia bombi* (A) and *C. expoeki* (B) show the numbers of SNPs coming from Alaskan (light green) or Switzerland (green) strains or the numbers of SNPs present in both populations (orange).

For both species, the Alaskan strains contributed around two- thirds of the observed overall genome variability (Fig. 3). For example, 94,418 variable genome positions (of a total 165,124 positions in the *C. bombi* genome) came from SNP calling in 5 Alaskan strains, while only 53,861 variable positions were from 22 Switzerland strains.

The mitochondrial genome is usually considered to be more variable than its nuclear counterpart and thus is often used as a sensitive genetic marker for distinguishing phylogenetically related species. Surprisingly, the mitochondrial genomes of Switzerland populations of both *Crithidia* spp. analyzed in this study have a very low SNP density (about 8,000 nt) and, as such, are even more conserved than nuclear genomes of the same strains. Of note, this analysis was performed using only coding regions of maxicircles and ignoring so-called divergent regions (31). For *C. expoeki*, the Alaskan strains have higher SNP densities in the mitochondrial genome (about 500 nt) compared to that of the nuclear genome. For most Alaskan strains of *C. bombi* SNP density is only slightly increased relative to that in the Switzerland population. Only one strain (AK08_053) exhibits the highest SNP density of 1 per 207 nt. This strain is also the most divergent by markers from the nuclear genome.

### Phylogenetic analysis.

**FIG 4:**
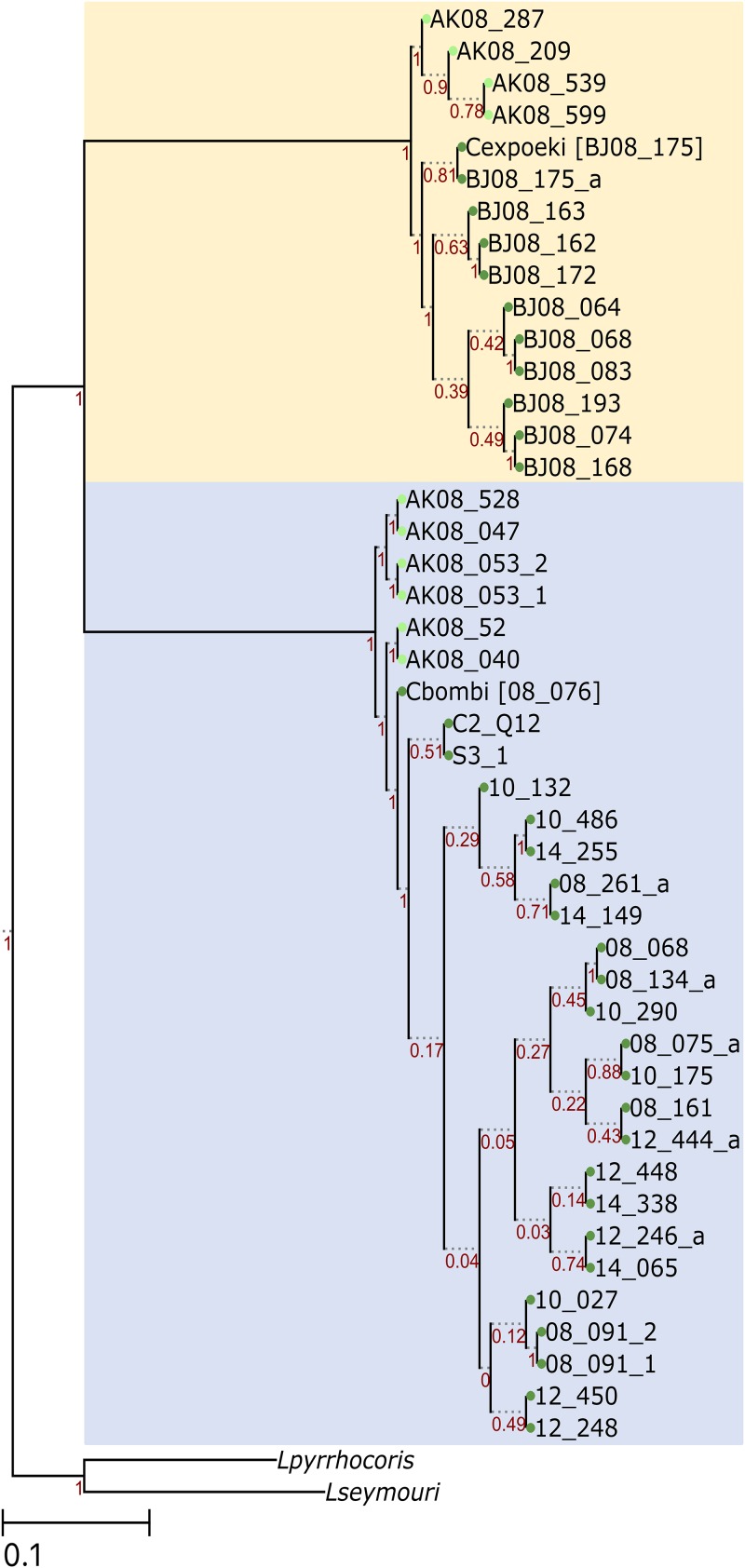
Phylogenetic analyses. A RaxML phylogenetic tree of two studied *Crithidia* strains is presented. *Leptomonas pyrrhocoris* H10 and *L. seymouri* were used as outgroups. *Crithidia bombi* and *C. expoeki* strains are on blue and moccasin backgrounds, respectively. The nodes of Alaskan and Switzerland strains are marked with light and dark green circles, respectively.

A whole-genome-level phylogenetic analysis was done by building a phylogenetic tree based on a set of 2,876 protein-coding genes (Fig. 4). All four used algorithms produced trees with similar topology. A few algorithm-dependent branch rearrangements were noted within Switzerland and Alaskan clades of both species, but the following conclusions can be drawn based on all four tree-building algorithms: (i) the Alaskan strains occupy a basal position on the tree; (ii) the type strains are basal to all other Switzerland strains; (iii) two *C. bombi* strains (C2_Q12 and S3_1, collected in Corsica and Sardinia) always form a clade, basal to all Switzerland strains, but the type strain B08_176 (a possible artifact of a reference mapping bias due to low SNP); and (iv) low bootstrap support values and topology dependency on the algorithm used within Alaskan and Switzerland clades of both *Crithidia* spp. indicate that these strains are very close and hard to resolve. A basal position of the Alaskan strains of both species may provide a hint regarding the origin of these strains.

### Copy number variation.

**FIG 5:**
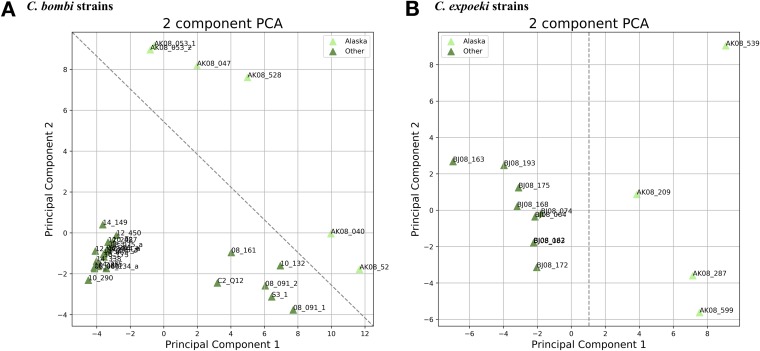
PCA for CNV analysis. A scatterplot of *Crithidia bombi* (A) and *C. expoeki* (B) strains shows the first and second principal components of PCA based on results of CNV analysis. Alaskan and Switzerland strains are marked with light and dark green triangles, respectively.

A copy number variation analysis of *C. bombi* and *C. expoeki* strains revealed 306 and 259 genome loci with CNVs, respectively. Locus deletions were as common as locus multiplications, and CNVs were rarely associated with gene boundaries, most of them not overlapping with genes. Principal component analyses (PCAs) demonstrated that the Alaskan strains can be easily separated from the Switzerland strains and tend to be more scattered on the planes of the first and second PCA axes (Fig. 5). In this rendering, the Switzerland strains of *C. bombi* formed two clusters. Whereas most strains were compactly clustered together, forming a first cluster, another small group of strains (08_161, 10_132, 08_091, S3_1, and C2_Q12) formed a separated second cluster. Notably, the Mediterranean strains (S3_1 and C2_Q12) also belonged to this second cluster (Fig. 5A). As with SNP density, the overall amount of variation between the strains was greater for the Alaskan strains.

### Signatures of selection and GO enrichment.

The nonsynonymous (*dN*) and synonymous (*dS*) substitution rates were estimated for nested M8 versus M7 models and revealed 990 and 430 genes with signs of positive selection for *Crithidia bombi* and *C. expoeki*, respectively.

GO enrichment analysis results are summarized in Table S6. Positively selected genes (Table S6, parts A and B) were enriched with GO terms that are associated with core genome maintenance. These are likely involved in DNA replication, chromosome segregation, and DNA reparation processes. In particular, the GO definitions “DNA metabolic process,” “microtubule-based movement,” “transcription, DNA-templated,” and “microtubule motor activity” were significantly enriched for both species, but the enrichment was more pronounced for *C. bombi*, probably due to the higher overall number of genes under positive selection. Note that “microtubular” genes are associated with the flagellum and its movement. Although not a proof, the importance of these genes was corroborated by a recent study showing that certain nectar compounds, taken up by the bees, can remove the flagellum and reduce the virulence of *C. bombi* (32).

We also analyzed an enrichment in gene lists based on Alaska-specific SNPs (Table S6, part D [*C. bombi*] and part I [*C. expoeki*]), Switzerland-specific SNPs (Table S6, part C [*C. bombi*] and part F [*C. expoeki*]), and SNPs from both populations (Table S6, part E [*C. bombi*] and part J [*C. expoeki*]). The last group is composed of genes that evolve in all strains of *Crithidia* spp. and therefore should reflect common pattern of genome evolution of that species. This enrichment pattern was similar for *C. bombi* and *C. expoeki* and resembles the overall pattern observed for positively selected genes, indicating the importance of genes involved in DNA repair, DNA replication, chromosome segregation, and cell division processes. The list of enriched genes according to the common SNP analysis largely overlaps the list of positively selected genes according to the M8/M7 LRT analysis.

The enrichment pattern was more complex when we compared lists based on Switzerland-specific and Alaska-specific SNPs only. Alaska-specific *C. bombi* genes showed marginal enrichment with GO-terms connected with the metabolism of fatty acids and genome maintenance, whereas in the Switzerland populations there was a strong enrichment of genes connected with “oxidation-reduction process” and “oxidoreductase activity.” For *C. expoeki*, Alaska-specific genes showed no terms connected with genome maintenance; instead, they were slightly enriched with genes involved in cytochrome complex assembly. Switzerland-specific genes were highly enriched with terms connected to the mitochondrial respiratory chain function (“NADH dehydrogenase activity,” “ubiquinone biosynthetic process,” and “mitochondrial electron transport”). We therefore propose that the evolution of genes involved in mitochondrion electron transport chain functioning is a specific feature of Switzerland populations of both species.

We also analyzed a selected set of genes (*gp63* and *amastins*) from the previous study (30) and found that none of the *gp63*-annotated genes was under positive selection. Three *amastins* from *C. expoeki* (Ce.1.12300, Ce.1.52410, and Ce.1.71020) were under positive selection, and the respective sites with an omega of >1 were Alaska specific. None of the *C. bombi amastins* appeared to be under positive selection.

## DISCUSSION

The intestinal parasite of the genus *Crithidia* and their social bumble bee hosts have been studied for many years with respect to behavior, ecology, and genetics (29, 33–35). This host-parasite interaction is characterized by a strong parasite-versus-host colony genotype interaction effect on infection success, such that only a few parasite strains (genotypes) are able to infect a given host colony and, vice versa, a colony is refractory to a range of strains and will only succumb to a few (18, 19, 25, 34). Such host-parasite systems tend to diversify genetically, and we demonstrate here that different strains of *Crithidia bombi* and *C. expoeki* vary considerably in terms of SNP and CNV. We showed that the SNP density is between 1,300 and 1,800 nt for *C. bombi* and ∼5,500 nt for *C. expoeki*; it is usually higher in Alaskan strains of both species. These values fit well in the range, known from other trypanosomatids (36, 37). *Crithidia bombi* proved to be more variable also when comparing the average number of variants per strain (6,116 versus 3,409 in *C. expoeki*). Most of the variants in *C. bombi* were found in the Alaskan samples. Interestingly, on a per-strain basis, *C. expoeki* (variations at 18.5 genomic loci/strain) contained more sites with CNV (gene duplications and gene losses) than *C. bombi* (11.3 genomic loci/strain). Taken together, the average genomic distance among strains was higher in the Alaskan samples than in those from the Central Europe, and this was the case for both *Crithidia* spp. (Fig. 5).

The differences between the two locations (Alaska and Central Europe [combining Switzerland and the Mediterranean]) are the most striking. The two cases of genomic variation analyzed here (SNP and CNV) result from mutation events changing single positions or leading to gene duplication or losses. On the other hand, the standing variation, observed on a population level, typically reflects the rate at which new variants emerge versus the rate, at which they are removed (or maintained) by selection. In fact, CNV has been suggested to be adapted to different local selection regimes in *Leishmania* (38) or in trypanosomatids in general (39). The actual selection pressure remains unknown in most cases, but copy numbers determine the level of gene expression. This trait is particularly relevant because of the peculiarities of the genome organization and gene expression in trypanosomatids (31). Previous work on *C. bombi* has demonstrated that the genotypic background of different host colonies exerts selection on which the parasite genotype is able to persist in the host and eventually get transmitted (27, 40), and a part of this variation is due to expression differences (22). Thus, we must assume that selection is an essential process determining the standing genomic variation of the natural parasite populations. Furthermore, with a more variable genetic background of a host, the parasite population is likely to become more genetically diverse as well. Some evidence for such an effect came from the field studies of *Crithidia bombi* and its host communities, where the host species identity was a significant factor, facilitating the distribution of strains among hosts (28). Host species also affected growth dynamics of the parasites and the seasonality of their epidemics, yielding a given diversity of the strains (29). As such, selection by a diverse host community may be one factor that could explain the higher parasite diversity in Alaskan populations (where there is higher host species diversity) versus Central European populations (where there is lower host species diversity). In addition, one should consider an effect of different ecological factors in these two regions. Host populations are less dense in Alaska than in Central Europe. Consequently, the infection prevalence is also lower in the former (ca. 6%) than in the latter (ca. 33%), simply because there are fewer opportunities to transmit when hosts are rarer. This also means that coinfections of a single host individual by more than one parasite genotype (and also the opportunities to exchange genes among genotypes of *Crithidia*) is lower in Alaska than in Central Europe. In line with this, the effect of niche overlap (the degree to which bees of different species utilize the same flowering plant species) is also different. Niche overlap is known to play a particularly important role for the genetic structure of the infecting parasite population in a given host community (28). In fact, transmission among host individuals from the same or different species is known to occur when they visit the same flower (13). However, even when the same flower is visited, the likelihood of picking up an infection decreases over time since the parasites were last deposited by an infected bee (18). This “waiting time” is much higher in low-density host populations. Thus, the role of a niche overlap for transferring an infection is less prominent in Alaska. Taken together, the diversifying selection by variable hosts and ecological factors suggests that parasite strains in the Alaskan populations should be more separated from each other (Fig. 5) and more variable as a whole (Fig. 3), which was confirmed in the present study.

The population structures of *C. bombi* and *C. expoeki* are typical for most trypanosomatids; i.e., they are somewhere on a continuum of freely mixing, sexual and clonal populations (41, 42). High rates of genetic exchange, and thus sexual reproduction with segregation according to the Mendelian rules, leading to the emergence of novel genotypes, was demonstrated for *C. bombi* (23). Based on some earlier observations, *Crithidia expoeki* is suspected to be more clonal than *C. bombi*, which would fit the overall differences between species observed in this work. Because genetic exchange (which primarily affects the nuclear genome) must be less common in the Alaskan populations, and thus populations will be more clonal, this might also help to explain why the variation in mitochondrial genome was lower than that of its nuclear counterpart in the high-transmission, more sexual populations of Central Europe, compared to the low-transmission, more clonal populations of Alaska. More evidence is needed to establish the causes and consequences of these processes with certainty.

Based on the genomic structure (Fig. 1), codon usage (Fig. 2), phylogenetic analyses (Fig. 4), and pattern of CNV (Fig. 5), the status of *C. expoeki* types as separate species is confirmed in our current work. At the same time, the Alaskan populations appear to be clearly separated from those of Central Europe, according to SNP and SNV data. Codon usage pattern, in contrast, does not separate Alaskan strains from the strains of Central Europe and appears to be a species-specific feature. Within each region and species, the phylogeny is flat, and clustering is also independent of the collection year. This pattern fits a scenario of rapid host-parasite coevolution, where the selective advantage of a given parasite strain is only temporary. Interestingly, parasite strains from Alaska, as well as the type strains of both species, appear to be basal in the rooted tree (Fig. 4). We have no ready explanation for the latter observation, since the type strains were chosen arbitrarily. In contrast, the more basal position of the Alaskan strains may reflect a genuine biological process. Given the close phylogenetic proximity of *Crithidia bombi* and *C. expoeki* to *Leptomonas* spp. (6, 30), it is plausible to suggest that these *Crithidia* strains entered the *Bombus* host via horizontal transfer from other insects that happened to visit the same plants, just as is observed today with spillovers of parasites from bumblebees to honeybees (43). Of note, mixed infections (facilitating horizontal transfer) are very common in trypanosomatids (26, 44–46). To elaborate a scenario, the *Bombus* clade has emerged in the mountains of Western China approximately 25 Mya ago, in a period of global cooling at the Eocene-Oligocene boundary (47). These insects spread to the East into the Americas and to the West into Europe. Although we have no definitive information on the age of the bumblebee-infecting *Crithidia*, it is possible that they have entered their new host group during the Eastern expansion of the bumblebees, which would render the Alaskan populations basal, as observed here.

It has been postulated that nuclear genome is more conserved compared to its mitochondrial counterpart, probably due to a higher overall mutation rate, determined by the presence of reactive oxygen species. However, this rule is not without exceptions in several lineages of eukaryotes, most prominently, in yeasts (48, 49).

Our study identified a number of genes with a signature of positive selection (Table S6). Several identified categories, such as DNA repair, DNA replication, or chromosome segregation, implicate cell division. The doubling time of *C. bombi* is on the order of 10 to 16 h (50), and a such fast-growing strain can be transmitted to a new host within a few days after infection (51). In culture, a faster-dividing strain almost invariably outgrows its slower competitors (44), but the outcome *in vivo* often depends on the host (26). This implies that other than speed of multiplication factors may influence a parasite’s survival in insects. For example, an improved metabolic performance is likely to add to the competitiveness against other strains. These GO-terms are enriched in the Swiss populations, where multiple infection and thus competition among strains within a host is more common than in the Alaska setting. As was implicitly assumed above, selection appears to play a more important role in the Alaska populations than the genetic exchange. In line with this argument, three *amastins*, genes encoding surface proteins implicated in the parasite-host interaction, seem to have originated in the Alaskan populations of *C. expoeki* and were presumably kept under the positive selection pressure.

In summary, our analyses of genomic variation among strains of the bumblebee-infecting *Crithidia* spp. give helpful insights into possible scenarios of selection and diversification of trypanosomatid parasites.

## MATERIALS AND METHODS

### Sample collection and whole-genome sequencing.

*Crithidia* spp. were examined for the presence of trypanosomatids (52) either during field trips at various sites or in the context of sampling spring queens for experimental work in the Zürich lab (Table S1). Samples were either collected and frozen as described previously (11) or collected from fresh feces and processed immediately. In each case, samples were submitted to fluorescence-activated cell sorting to differentiate single cells and produce clonal lines, which were analyzed further (50). Libraries were produced using an Illumina TruSeq kit and sequenced on the Illumina HiSeq 2500 and HiSeq 4000 platforms (Illumina, San Diego, CA) at the Functional Genomic Center, Zürich, Switzerland (Table S1).

### Read mapping and processing.

Reads were trimmed for quality and sequencing adaptors with Trimmomatic v.0.36 (53) and quality controlled using FastQC v.0.11.8 (54). Trimmed reads were mapped with Bowtie2 v.2.3.4.1 (55) using the “–very-sensitive,” “–end-to-end” options. Downstream processing was done with SAMtools v.1.9 (56, 57) and in-house Python scripts or Linux core utilities (*grep*, *awk*, *sort*, and *uniq*). Uniquely mapped reads were selected for further analyses with *grep* (picking properly aligned pairs by searching the “YT:Z:CP” pattern and discarding reads with secondary alignments by discarding lines with the “XS:i:” pattern), and PCR duplicates were removed with SAMtools. Final sorted .bam files with correctly mapped unique read pairs were used for all downstream analyses.

### SNP calling.

Variant calling was done using bcftools*/*SAMtools v1.9. Only SNPs were taken for analysis; indels were filtered out with the “–remove-indels” option. The resulting VCF was used to build distance matrices and generate consensus fasta files using the VCF consensus tool. Gene sequences with SNPs typical for each strain were extracted with a Python script using gene coordinates from the gff annotation (PRJEB21109 and PRJEB21108). Alaska-specific and Switzerland-specific SNPs were determined with a Python script: SNP was counted as Alaskan specific if it was observed only in one or more Alaskan strains but not in any Switzerland strain (and the reverse was used for Switzerland-specific positions). Genes that contain only Alaska-specific SNPs or only Switzerland-specific SNPs were determined with BEDTools v.2.16.2 (58) and an in-house Python script.

### Reference-free SNP calling and clustering.

DiscoSnp++ (59) was used to obtain SNPs for all sequenced strains (and all strains including previously sequenced type strains 08_076 and BJ08_175 in a separate run) with an algorithm that does not rely on genome reference assembly and therefore can compare samples from different species. Single-end reads were used for 08_076, BJ08_175 (PRJEB21109 and PRJEB21108), and paired-end reads generated in frame of this study were used for all other strains.

### Maxicircle assembly.

Sequences of the mitochondrial maxicircles of *C. bombi* and *C. expoeki* were assembled from PacBio sequencing data (PRJEB21109 and PRJEB21108), which were used previously for nuclear genome assembly (30). A subset of reads with BLASTN hits to *12s* or *nd5* genes was compiled from the total PacBio data set. This subset was then assembled with the Canu assembler v1.8 (60). Full-length circular molecule was obtained for *C. expoeki*, for which numerous of reads, matching our subsetting criteria, were found. For *C. bombi*, the overall read coverage of maxicircle was much lower and, thus only coding regions with short flanks were assembled. Assembly quality was checked with Illumina paired-end reads mapping (mapping with Bowtie2, average insert size plotting with a Python script). Coding regions were annotated with BLASTN, followed by manual alignment curation of *12s* and *nd5* gene sequences. Coding regions with 500-nt flanks were extracted and used for read mapping. Reads were mapped and processed as described above using the pipeline for the nuclear genome.

### Copy number variation analysis.

Copy number variation analysis was done with the cn.MOPS package for R (61). Segmentation algorithm was “DNAcopy,” and window lengths were 200, 300, 400, or 500 nt. Window length did not change the results greatly, so we selected “WL = 300 nt” for the final analysis. Regions with significant CNV detected were summarized in Tables S2 to S4. A value of 2 corresponds to the normal diploid locus. A value of 1 indicates the loss of a single allele, and a value of 0 signifies the complete loss of this locus. Values of >2 correspond to possible locus multiplications. The states of all detected variable loci were used as feature vectors for clustering and PCA (performed in Python, scikit-learn, and Seaborn packages).

10.1128/mSphere.00482-19.3 TABLE S3 Copy number variations in *C. bombi*. Copy number variations detected in genome loci of C. *bombi* (cn.MOPS package, WL = 300). Each cell’s value shows the relative copy number of given genomic locus. A value of 2 corresponds to normally diploid state. Values of greater than 2 correspond to locus multiplication events. A value of 1 to loss of one allele and 0 denotes absence of locus in given genome. The last column shows if any gene annotations overlap (or partially overlap) with given locus. Download Table S3, PDF file, 0.8 MB. Copyright © 2019 Gerasimov et al. 2019 Gerasimov et al. This content is distributed under the terms of the Creative Commons Attribution 4.0 International license.

10.1128/mSphere.00482-19.4 TABLE S4 Copy number variations in *C. expoeki*. Copy number variations were detected in the genome loci of C. *expoeki* (cn.MOPS package, with WL = 300). Download Table S4, PDF file, 0.3 MB. Copyright © 2019 Gerasimov et al. 2019 Gerasimov et al. This content is distributed under the terms of the Creative Commons Attribution 4.0 International license.

### Phylogenetic analysis.

Orthologous groups (OGs) of genes were taken from (30). A total of 2,876 OGs containing a single gene per species (1-to-1 orthologues) were selected for this analysis. Two data sets were compiled to build the tree. The first set included all studied *Crithidia* strains, along with the two phylogenetically related species, *Leptomonas seymouri* (44) and *Leptomonas pyrrhocoris* (36), to root the tree. This data set is referred to as “Lept.” The second set (referred to as “Tryp”) also included *Blechomonas ayala*, *Crithidia fasciculata*, and Trypanosoma brucei as outgroup species (see references 30 and 62 for details on the outgroups). For the Tryp and Lept data sets, the orthologues for each gene were aligned with mafft v.7.305 (63) with default parameters. These alignments were combined into multilocus alignments and treated with Gblocks v.0.91b (64, 65). Phylogenetic trees were built with the “ETE3 build” from the ETE3 package v.3.1.1 (66) using PhyML (67), RAxML (68), BIONJ (69), and FastTree (70) algorithms. Topology of the other obtained trees was compared to that tree and is accessible in Newick format in Table S5.

10.1128/mSphere.00482-19.5 TABLE S5 Phylogenetic analysis results. Tree topologies obtained in phylogenetic analysis are summarized in this table in Newick format (with branch length and bootstrap support value if available). Trees were built for two datasets Lept and Tryp. Lept dataset included sequences for *Crithidia bombi* strains, *C. expoeki* strains, *Leptomonas seymouri*, and *L. pyrrhocoris* H10. The Tryp dataset also included sequences for *Blechomonas ayala*, Trypanosoma brucei, and *Crithidia fasciculata*. Download Table S5, XLSX file, 0.01 MB. Copyright © 2019 Gerasimov et al. 2019 Gerasimov et al. This content is distributed under the terms of the Creative Commons Attribution 4.0 International license.

For the final alignment of 2,876 genes, 3,795,880 positions (85% of the total alignment length) in 18,508 selected blocks for the Lept data set, and 3,185,151 positions (67%) in 34,891 selected blocks for the Tryp data set, were left after Gblocks polishing. Phylogenetic trees obtained for the Tryp data sets confirmed that two *Leptomonas* species are the closest outgroup for the *Crithidia* spp. studied here. In further analyses, the trees obtained from the Lept data set (based on a greater number of genomic loci) were used. From the four congruent trees, built with different algorithms, we present only the RAxML tree.

### Analysis of selection.

A total of 7,808 sets of aligned gene sequences for each strain and for each gene of *C. bombi*, as well as 7,851 comparable sets for *C. expoeki* strains, were prepared. The Ete3 python package as a wrapper around PAML’s codeml to search for selection was used. The best model was identified using likelihood ratio test (LRT) for the best-fitting model among pairs of nested models in two tests: (i) M2 versus M1 and (ii) M8 versus M7. Both models were site models and detected positive selection acting on sites. LRT results for M2 versus M1 and for M8 versus M7 were very similar; hence, we used the predictions made with M8 versus M7 for further discussions. For genes in which the LRT was significant, we looked at positively selected sites requesting a Bayes Empirical Bayes posterior probability of >0.95.

### GO enrichment.

GO enrichment on gene subsets was calculated with the top.GO v.1.0 R package (71). The GO enrichment was performed for subsets of genes that were found to be positively selected in *C. bombi* and in *C. expoeki* strains.

Lists of genes carrying only Alaska-specific SNPs, only Switzerland-specific SNPs, and both types of SNPs were produced. These gene sets were used to discriminate between genes that evolve only in Alaskan strains, genes that evolve only in Switzerland strains (with respect to type strain), and genes that evolve both in Alaskan population and in Switzerland population but have different patterns of nucleotide changes. Top.GO software was used to demonstrate functional enrichment in genes. Enriched GO categories and their definitions are listed in Table S6.

10.1128/mSphere.00482-19.6 TABLE S6 GO enrichment analysis. GO enrichment detected by top.GO. (A) Enrichment in list of genes under positive selection in *C. bombi.* (B) Enrichment in list of genes under positive selection in *C. expoeki.* (C) Enrichment in list of genes that have SNPs only in Switzerland strains of *C. bombi.* (D) Enrichment in list of genes that have SNPs only in Alaskan strains of *C. bombi.* (E) Enrichment in list of genes that have SNPs in all strains of *C. bombi.* (F) Enrichment in list of genes that have SNPs only in Switzerland strains of *C. expoeki*. (I) Enrichment in list of genes that have SNPs only in Alaskan strains of *C. expoeki*. (J) Enrichment in list of genes that have SNPs in all strains of *C. expoeki*. Values in ‘Annotated’, ‘Expected’, and ‘Significant’ columns are numbers of genes with that GO annotation. Download Table S6, PDF file, 0.8 MB. Copyright © 2019 Gerasimov et al. 2019 Gerasimov et al. This content is distributed under the terms of the Creative Commons Attribution 4.0 International license.
