# Supplementary material for: Genomic Variation among Strains of Crithidia bombi and C. expoeki
Source: mSphere. 2019 Sep 11;4(5):e00482-19. doi: 10.1128/mSphere.00482-19 (PMC6739494; doi:10.1128/mSphere.00482-19)
Supplement: TABLE S1 [file mSphere.00482-19-st001.docx]

| **Code #^1^** | ***Crithidia* type^2^** | **Host species and caste^3^** | **Collection site** | **Date collected** |
| --- | --- | --- | --- | --- |
| 08_068 | A1 | *Bombus* *terrestris*, Q | CH, Neunforn | 31.3.2008 |
| 08_075 | A1 | *B. terrestris*, Q | CH, Neunforn | 31.3.2008 |
| **08_076** | A1 | *B. terrestris*, Q | CH, Neunforn | 31.3.2008 |
| 08_091 | A1 | *B. terrestris*, Q | CH, Neunforn | 31.3.2008 |
| 08_134 | A1 | *B. terrestris*, Q | CH, Neunforn | 31.3.2008 |
| 08_161 | A1 | *B. terrestris*, Q | CH, Neunforn | 31.3.2008 |
| 08_261 | A1 | *B. terrestris*, Q | CH, Neunforn | 2.4.2008 |
| **BJ08_175** | B1 | *Bombus lucorum,* W | CH, Röschenz | 9.6.2008 |
| 10_027 | A1 | *B. terrestris*, Q | CH, Neunforn | 25.3.2010 |
| 10_132 | A1 | *B. terrestris*, Q | CH, Aesch | 29.3.2010 |
| 10_175 | A1 | *B. terrestris*, Q | CH, Aesch | 29.3.2010 |
| 10_290 | A1 | *B. terrestris*, Q | CH, Neunforn | 29.3.2010 |
| 10_489 | A1 | *B. terrestris*, Q | CH, Neunforn | 14.4.2010 |
| 12_246 | A1 | *B. terrestris*, Q | CH, Neunforn | 21.3.2012 |
| 12_248 | A1 | *B. terrestris*, Q | CH, Neunforn | 21.3.2012 |
| 12_444 | A1 | *B. terrestris*, Q | CH, Aesch | 23.3.2012 |
| 12_448 | A1 | *B. terrestris*, Q | CH, Neunforn | 23.3.2012 |
| 12_450 | A1 | *B. terrestris*, Q | CH, Neunforn | 23.3.2012 |
| 14_065 | A1 | *B. terrestris*, Q | CH, Neunforn | 10.4.2014 |
| 14_149 | A1 | *B. terrestris*, Q | CH, Neunforn | 11.4.2014 |
| 14_255 | A1 | *B. terrestris*, Q | CH, Aesch | 12.4.2014 |
| 14_338 | A1 | *B. terrestris*, Q | CH, Aesch | 12.4.2014 |
| AK08_040 | A1 | *Psythirus* *insularis*, Q | Alaska, Willow Creek | 10.6.2008 |
| AK08_047 | A1 | *P. insularis*, Q | Alaska, Willow Creek | 14.6.2008 |
| AK08_052 | A1 | *P. insularis*, Q | Alaska, Willow Creek | 14.6.2008 |
| AK08_053 | A2 | *P. insularis*, Q | Alaska, Willow Creek | 14.6.2008 |
| AK08_209 | B2 | *Bombus* *flavifrons*, Q | Alaska, Delta Junction | 16.6.2008 |
| AK08_287 | B2 | *B. flavifrons*, Q | Alaska, Delta Junction | 17.6.2008 |
| AK08_528 | A1 | *B. lucorum*, Q | Alaska, Anchor Point | 27.6.2008 |
| AK08_539 | B2 | *Bombus melanopygus*, W | Alaska, Anchor Point | 30.6.2008 |
| AK08_599 | B2 | *B. melanopygus*, Q | Alaska, Moose Pass | 1.7.2008 |
| BJ08_064 | B1 | *B. lucorum*, W | CH, Movellier | 9.6.2008 |
| BJ08_068^4^ | B1 | *Bombus pratorum*, W | CH, Movellier | 9.6.2008 |
| BJ08_074 | B1 | *B. lucorum*, W | CH, Movellier | 9.6.2008 |
| BJ08_083 | B1 | *B. pratorum*, W | CH, Movellier | 9.6.2008 |
| BJ08_162 | B1 | *B. pratorum*, W | CH, Röschenz | 9.6.2008 |
| BJ08_163 | B1 | *B. pratorum*, W | CH, Röschenz | 9.6.2008 |
| BJ08_168 | B1 | *B. pratorum*, W | CH, Röschenz | 9.6.2008 |
| BJ08_172 | B1 | *B. pratorum*, W | CH, Röschenz | 9.6.2008 |
| *BJ08_175a* | B1 | *B. lucorum*, W | CH, Röschenz | 9.6.2008 |
| BJ08_193 | B1 | *B. pratorum*, W | CH, Röschenz | 9.6.2008 |
| C2_Q12 | A1 | *B. terrestris*, Q | Corsica | 10.2.2012 |
| S3_1 | A1 | *B. terrestris*, W | Sardegna | 16.2.2012 |
