# Supplementary material for: Genomic Variation among Strains of Crithidia bombi and C. expoeki
Source: mSphere. 2019 Sep 11;4(5):e00482-19. doi: 10.1128/mSphere.00482-19 (PMC6739494; doi:10.1128/mSphere.00482-19)
Supplement: TABLE S2 [file mSphere.00482-19-st002.pdf]

Strain description		Sequencing stats					SNP calling stats							SNP in maxicircle							Overall genome variation	
Strain code	Species	Sequencing platform	Total reads, M	Proper pairs mapped	Average coverage	Median coverage	SNP, reference	SNP, homozygote	SNP, heterozygote	Transitions	Transversions	Ts/Tv	SNPs, %	Average coverage	Median coverage	SNP, reference	SNP, homozygote	SNP, heterozygote	Transitions	Transversions	SNP density, nt	1 / SNP density
08 068	*bombi*	Hiseq 2500	32.43	93.39%	123.1	110	148461	6490	10173	11771	4892	2.41	0.101	194.7	213	86	2	0	2	0	1923	
08 075 a	*bombi*	Hiseq 2500	33.15	94.96%	127.7	115	147717	6464	10943	12283	5124	2.40	0.105	718.5	792	84	3	1	3	1	1841	
08 091 1	*bombi*	Hiseq 4000	23.42	87.21%	110.2	107	148528	6271	10325	11666	4930	2.37	0.101	377.7	387	82	3	3	5	1	1930	
08 091 2	*bombi*	Hiseq 4000	20.61	88.31%	97	94	148507	6244	10373	11676	4941	2.36	0.101	325.7	333	83	3	2	5	0	1928	
08 134 a	*bombi*	Hiseq 2500	24.6	94.52%	94.3	82	148281	6457	10386	11893	4950	2.40	0.102	471.0	516	86	2	0	2	0	1902	
08 161	*bombi*	Hiseq 4000	14.13	88.12%	66.9	60	147737	6293	11094	12343	5044	2.45	0.105	500.0	521	83	3	2	5	0	1843	
08 261 a	*bombi*	Hiseq 2500	23.7	94.18%	90	82	146715	6224	12185	13095	5314	2.46	0.111	640.7	697	86	1	1	1	1	1740	
10 027	*bombi*	Hiseq 2500	26.56	93.80%	101.6	92	147898	6215	11011	12220	5006	2.44	0.104	118.5	125	85	3	0	3	0	1860	
10 132	*bombi*	Hiseq 4000	23.39	87.33%	110.6	105	147504	6328	11292	12469	5151	2.42	0.107	316.0	322	84	1	3	3	1	1818	
10 175	*bombi*	Hiseq 2500	31.03	94.47%	119	109	147507	5976	11641	12467	5150	2.42	0.107	93.5	97	85	3	0	3	0	1819	
10 290	*bombi*	Hiseq 2500	30.66	93.17%	117.2	103	147823	6291	11010	12221	5080	2.41	0.105	167.9	192	84	3	1	3	1	1852	
10 486	*bombi*	Hiseq 2500	33.57	93.74%	129.2	118	147350	6365	11409	12560	5214	2.41	0.108	184.8	209	86	2	0	1	1	1803	
12 246 a	*bombi*	Hiseq 2500	25.09	95.24%	96.2	85	147432	6157	11535	12541	5151	2.43	0.107	474.3	516	85	2	1	2	1	1811	
12 248	*bombi*	Hiseq 2500	42.04	94.96%	162.4	148	147680	6341	11103	12305	5139	2.39	0.106	114.3	127	85	3	0	3	0	1837	
12 444 a	*bombi*	Hiseq 2500	27.49	95.01%	106.1	94	148058	6101	10965	12091	4975	2.43	0.103	536.0	610	87	1	0	1	0	1877	
12 448	*bombi*	Hiseq 2500	25.86	92.59%	98.2	90	147426	6328	11370	12526	5172	2.42	0.107	85.9	89	87	1	0	1	0	1810	
12 450	*bombi*	Hiseq 2500	27.12	93.26%	103.6	95	147239	6083	11802	12679	5206	2.44	0.108	113.8	117	85	3	0	3	0	1791	
14 065	*bombi*	Hiseq 2500	29.92	95.64%	116.1	103	147671	6315	11138	12389	5064	2.45	0.106	96.7	106	87	1	0	1	0	1836	
14 149	*bombi*	Hiseq 2500	22.81	93.59%	87.5	80	146487	6383	12254	13201	5436	2.43	0.113	69.4	70	86	2	0	2	0	1719	
14 255	*bombi*	Hiseq 2500	22.94	92.02%	86.2	80	147029	6335	11760	12811	5284	2.42	0.110	67.2	68	87	1	0	1	0	1771	
14 338	*bombi*	Hiseq 2500	21.41	92.47%	81.1	71	147666	6510	10948	12394	5064	2.45	0.106	128.9	125	85	2	1	2	1	1835	
AK08 040	*bombi*	Hiseq 4000	20.74	86.27%	98.1	89	140831	10880	13413	17286	7007	2.47	0.147	187.1	191	79	8	1	8	1	1319	
AK08 047	*bombi*	Hiseq 4000	21.1	88.89%	100.1	93	88627	9060	67437	55992	20505	**2.73**	0.463	302.3	304	79	9	0	8	1	419	
AK08 053 1	*bombi*	Hiseq 4000	23.89	88.67%	113.5	103	81103	64085	19936	61554	22467	**2.74**	0.509	381.6	404	12	76	0	69	7	381	
AK08 053 2	*bombi*	Hiseq 4000	20.23	89.69%	96.2	87	81183	63426	20515	61495	22446	**2.74**	0.508	357.3	371	12	76	0	69	7	382	
AK08 528	*bombi*	Hiseq 4000	25.79	87.74%	122.2	118	88825	9780	66519	55853	20446	**2.73**	0.462	310.1	298	78	9	1	8	2	420	
AK08 52	*bombi*	Hiseq 4000	20.87	86.38%	98.6	89	140166	10721	14237	17738	7220	2.46	0.151	331.7	336	79	8	1	7	2	1284	
C2 Q12	*bombi*	Hiseq 4000	22.74	88.52%	107.6	99	148488	7220	9416	11712	4924	2.38	0.101	150.5	143	83	4	1	3	2	1926	
S3 1	*bombi*	Hiseq 4000	20.45	88.75%	96.7	88	147171	6221	11732	12735	5218	2.44	0.109	141.5	136	82	3	3	5	1	1785	
AK08 209	*expoeki*	Hiseq 4000	15.53	90.98%	68.8	77	19910	20526	7289	19988	7827	**2.55**	0.583	643.9	793	19	36	2	30	8	1225	
AK08 287	*expoeki*	Hiseq 4000	17.93	88.47%	78.8	88	19659	20523	7543	20228	7838	**2.58**	0.588	1004.3	1248	18	38	1	31	8	1214	
AK08 539	*expoeki*	Hiseq 4000	20.22	87.50%	88.6	99	19993	20102	7630	19902	7830	**2.54**	0.581	560.5	692	20	36	1	29	8	1229	
AK08 599	*expoeki*	Hiseq 4000	18.04	87.56%	79.3	89	19880	20210	7635	19981	7864	**2.54**	0.583	812.0	994	20	36	1	29	8	1224	
BJ08 064	*expoeki*	Hiseq 4000	16.97	88.28%	74.3	82	41626	1930	4169	4367	1732	**2.52**	0.128	1038.0	1120	52	4	1	5	0	5587	
BJ08 068	*expoeki*	Hiseq 4000	11.7	81.06%	51.3	51	41804	1969	3952	4177	1744	2.40	0.124	79.1	67	52	2	3	5	0	5755	
BJ08 074	*expoeki*	Hiseq 4000	18.31	86.48%	79.9	87	41561	1861	4303	4395	1769	2.48	0.129	1284.6	1322	56	1	0	1	0	5529	
BJ08 083	*expoeki*	Hiseq 4000	17.07	92.13%	75.2	84	41798	2001	3926	4185	1742	2.40	0.124	1058.3	1137	51	4	2	6	0	5750	
BJ08 162	*expoeki*	Hiseq 4000	18.53	88.76%	81.1	91	41795	1932	3998	4223	1707	2.47	0.124	1399.1	1466	56	1	0	1	0	5747	
BJ08 163	*expoeki*	Hiseq 4000	16.54	83.74%	72.2	80	41596	1984	4145	4372	1757	2.49	0.128	191.2	140	49	8	0	6	2	5560	
BJ08 168	*expoeki*	Hiseq 4000	22.38	86.88%	97.7	110	41530	2041	4154	4414	1781	2.48	0.130	878.2	944	53	3	1	3	1	5501	
BJ08 172	*expoeki*	Hiseq 4000	15.56	85.15%	67.9	75	41804	1969	3952	4221	1700	2.48	0.124	806.5	867	56	1	0	1	0	5755	
BJ08 175a	*expoeki*	Hiseq 4000	15.63	88.14%	68.2	76	43466	347	3912	3023	1236	2.45	0.089	763.1	805	56	1	0	1	0	8001	
BJ08 193	*expoeki*	Hiseq 4000	15.48	86.55%	67.8	74	41468	1877	4380	4455	1802	2.47	0.131	325.5	245	51	6	0	6	0	5446	
