# Supplementary material for: Genomic Variation among Strains of Crithidia bombi and C. expoeki
Source: mSphere. 2019 Sep 11;4(5):e00482-19. doi: 10.1128/mSphere.00482-19 (PMC6739494; doi:10.1128/mSphere.00482-19)
Supplement: TABLE S3 [file mSphere.00482-19-st003.pdf]

chrom	start	end	width	08_068	08_075_a	08_091_1	08_091_2	08_134_a	08_161	08_261_a	10_027	10_132	10_175	10_290	10_486	12_246_a	12_248	12_444_a	12_448	12_450	14_065	14_149	14_255	14_338	AK08_040	AK08_047	AK08_053_1	AK08_053_2	AK08_52	AK08_528	C2_Q12	S3_1	Genes overlapping the region
ENAIOESO01	1	27000	27000	2	1	2	2	1	2	2	1	2	2	1	2	3	2	2	2	2	1	2	2	1	3	2	2	2	2	2	2	2	Cb.1.24370 (-); Cb.1.24420 (-); Cb.1.24260 (-);
ENAIOESO01	27301	28200	900	2	2	2	2	2	1	2	2	2	2	2	2	2	2	2	2	2	2	2	2	2	2	2	2	2	2	2	2	2	
ENAIOESO01	223501	224400	900	2	2	1	2	2	2	2	2	2	2	2	2	2	2	2	2	2	2	2	2	2	2	2	2	2	2	2	2	2	
ENAIOESO01	255001	255900	900	2	2	1	1	2	2	2	2	2	2	2	2	2	2	2	2	2	2	2	2	2	2	2	2	2	2	2	2	2	
ENAIOESO01	324001	327300	3300	2	2	3	2	2	2	2	2	2	4	3	2	2	2	3	2	2	3	2	2	3	2	2	1	1	2	2	2	2	Cb.1.24120 (+);
ENAIOESO01	168001	168900	900	2	2	2	2	2	2	2	2	2	2	2	2	2	2	2	2	2	2	2	2	2	2	2	2	2	2	2	1	2	
ENAIOESO01	49501	50400	900	2	3	2	2	2	2	2	2	2	2	2	2	2	2	2	2	2	2	2	2	2	2	2	2	2	2	2	2	2	Cb.1.05210 (-);
ENAIOESO01	61201	63000	1800	2	2	2	2	2	2	2	2	2	2	2	2	2	2	3	2	2	3	2	2	3	2	2	2	2	2	2	2	2	
ENAIOESO01	104701	105600	900	2	2	2	2	2	2	2	2	2	2	2	2	2	2	2	2	2	2	2	2	2	2	2	2	2	2	2	1	2	
ENAIOESO01	385501	387000	1500	2	2	2	2	2	2	2	2	2	2	2	2	2	2	2	2	2	2	2	2	2	2	2	2	2	2	4	2	2	
ENAIOESO01	482101	483600	1500	2	2	2	2	2	2	2	2	2	2	2	2	2	2	2	2	2	1	2	2	2	2	2	2	2	2	3	2	2	Cb.1.04000 (-);
ENAIOESO01	661801	667200	5400	2	2	2	2	2	2	2	2	2	2	2	2	3	2	2	2	2	2	2	2	2	2	2	2	2	2	2	2	2	Cb.1.03890 (-); Cb.1.05970 (-);
ENAIOESO01	814801	816000	1200	2	2	2	2	2	2	2	2	2	2	2	2	2	2	2	2	2	2	2	2	2	2	2	2	2	2	5	2	2	Cb.1.02730 (+);
ENAIOESO01	903001	904500	1500	1	2	2	2	2	2	2	2	2	2	2	2	2	2	2	2	2	2	2	2	2	2	2	2	2	2	2	2	2	Cb.1.03240 (+);
ENAIOESO01	1476601	1479300	2700	2	2	2	2	2	2	2	2	2	2	3	3	2	3	2	4	2	2	3	2	2	2	2	2	2	2	2	2	2	
ENAIOESO01	136801	137700	900	2	2	2	2	2	2	2	2	2	2	2	2	2	2	2	2	2	2	2	2	2	2	3	2	2	2	3	2	2	Cb.1.44530 (+);
ENAIOESO01	221101	223800	2700	2	2	2	2	2	2	2	2	2	2	4	2	2	2	2	2	2	2	4	2	2	2	4	2	4	2	4	2	2	Cb.1.44640 (+);
ENAIOESO01	380401	381600	1200	2	2	2	2	2	2	2	2	2	2	2	2	2	3	2	2	2	2	2	2	2	2	2	2	2	2	2	2	2	
ENAIOESO01	392701	394500	1800	2	0	2	2	3	2	3	3	2	2	3	2	2	1	2	0	3	2	2	4	2	2	2	2	2	2	2	2	2	
ENAIOESO01	543301	562500	19200	1	1	4	2	1	2	1	1	2	1	1	1	1	1	1	1	2	1	1	1	1	2	1	0	0	2	1	2	2	Cb.1.45750 (-); Cb.1.45870 (-); Cb.1.45930 (-); Cb.1.45980 (-); Cb.1.46000 (-); Cb.1.45360 (-); Cb.1.45900 (-);
ENAIOESO01	619501	620400	900	2	2	2	2	2	2	2	2	2	2	2	2	2	2	3	2	2	2	2	2	2	2	2	2	2	2	2	2	2	Cb.1.44400 (+);
ENAIOESO01	672301	674100	1800	2	1	2	2	2	2	2	2	2	2	2	1	2	2	2	2	2	2	2	2	2	2	2	2	2	2	4	3	2	Cb.1.44520 (+);
ENAIOESO01	678901	684300	5400	3	2	3	3	1	2	1	2	3	2	2	2	1	2	2	2	2	1	2	2	2	2	3	2	2	2	3	3	2	
ENAIOESO01	687001	691606	4606	3	2	2	2	2	2	2	2	3	2	2	2	2	2	2	2	2	2	2	2	2	2	2	1	1	2	2	3	2	
ENAIOESO01	120601	121500	900	2	2	5	2	2	5	2	2	5	2	2	2	2	2	2	2	2	2	2	2	2	5	2	2	2	5	5	5	5	Cb.1.14260 (+);
ENAIOESO01	133801	135000	1200	2	2	2	2	2	2	2	2	2	2	2	2	2	2	2	2	2	2	2	2	2	4	4	2	2	4	5	5	2	Cb.1.13780 (+); Cb.1.15100 (-); Cb.1.15720 (-);
ENAIOESO01	139501	141900	2400	4	2	2	2	4	5	2	2	3	2	4	2	2	2	2	2	2	4	2	2	2	4	2	2	2	4	2	3	2	Cb.1.12670 (+); Cb.1.15680 (-);
ENAIOESO01	342301	343200	900	2	2	2	2	2	1	2	2	2	2	2	2	2	2	2	2	2	2	2	2	2	2	2	2	2	2	2	2	2	
ENAIOESO01	451201	452100	900	2	2	2	1	2	2	2	2	2	2	2	2	2	2	2	2	2	2	2	2	2	2	2	2	2	2	2	2	2	
ENAIOESO01	508801	510000	1200	2	2	2	2	2	2	2	2	2	2	2	2	2	2	2	2	2	2	2	2	2	3	2	2	2	2	3	2	2	
ENAIOESO01	564901	565800	900	2	2	2	2	2	2	2	2	2	2	2	2	2	2	2	2	2	2	2	2	2	2	2	2	2	2	2	1	2	
ENAIOESO01	598801	600900	2100	2	3	2	3	2	3	3	3	2	2	3	3	4	2	1	2	2	2	3	2	2	1	1	2	2	1	1	4	2	Cb.1.15920 (-);
ENAIOESO01	894301	897300	3000	2	2	2	2	2	2	2	2	2	2	2	2	2	2	2	2	2	2	2	2	2	2	2	2	2	4	3	2	2	
ENAIOESO01	1043701	1044600	900	2	2	2	2	2	2	2	2	2	2	2	2	2	2	2	2	2	2	2	2	2	2	1	2	2	2	2	2	2	
ENAIOESO01	1126501	1127700	1200	2	2	2	2	2	2	2	2	2	2	2	2	2	2	2	2	2	2	2	2	2	2	2	2	2	3	1	2	2	Cb.1.15010 (-);
ENAIOESO01	1134001	1135200	1200	2	2	4	4	2	4	2	2	4	2	2	2	2	2	2	2	2	2	2	2	2	4	2	2	2	3	2	4	2	
ENAIOESO01	1166401	1167600	1200	2	2	2	2	2	2	2	2	2	2	2	2	2	2	2	2	2	2	2	2	2	2	3	2	2	2	4	2	2	Cb.1.15670 (-);
ENAIOESO01	1169701	1171200	1500	2	2	4	2	2	2	2	2	4	2	2	2	2	2	2	2	2	2	2	2	2	2	2	2	2	4	4	4	2	Cb.1.15670 (-);
ENAIOESO01	1	900	900	2	2	3	3	2	3	2	2	3	2	2	2	2	2	2	2	2	2	2	2	2	2	2	2	2	3	2	2	3	
ENAIOESO01	96601	97500	900	2	2	1	1	2	1	2	2	1	2	2	2	2	2	2	2	2	2	2	2	2	2	1	2	2	1	1	1	1	
ENAIOESO01	148201	149100	900	2	2	2	2	2	2	2	2	2	2	2	2	2	2	2	2	2	2	2	2	2	2	2	2	2	1	2	2	2	
ENAIOESO01	166501	167400	900	2	2	2	2	2	2	2	2	2	2	2	2	2	2	2	2	2	2	2	2	2	2	2	2	2	2	2	2	0	
ENAIOESO01	179401	180600	1200	2	2	2	2	2	2	2	2	2	3	2	2	2	2	2	2	2	2	2	2	2	2	2	2	2	2	2	2	2	Cb.1.57160 (+);
ENAIOESO01	251401	253800	2400	2	2	2	2	2	1	2	2	2	2	3	2	2	2	2	1	2	2	3	2	2	2	2	2	2	3	2	4	2	Cb.1.57030 (+);
ENAIOESO01	363301	368400	5100	2	2	3	3	2	2	1	1	3	2	2	2	2	2	2	2	2	2	2	2	2	3	3	3	2	3	3	2	2	Cb.1.43170 (+); Cb.1.43960 (-);
ENAIOESO01	1	3300	3300	2	2	2	2	2	2	2	2	2	2	2	2	3	2	3	3	2	2	3	2	3	2	2	2	2	2	2	2	2	Cb.1.34890 (-);
ENAIOESO01	6301	9600	3300	2	1	2	2	2	2	2	2	3	1	2	3	1	1	2	2	2	2	3	2	2	2	2	2	2	2	2	2	2	Cb.1.34740 (-);
ENAIOESO01	74101	75900	1800	2	2	2	2	2	2	2	2	2	2	1	2	2	2	2	2	2	0	2	2	2	2	2	2	2	2	2	2	2	
ENAIOESO01	601201	603300	2100	2	2	2	2	2	2	2	2	2	2	2	2	2	3	2	3	2	3	2	2	2	2	2	2	2	2	2	2	2	
ENAIOESO01	687001	689400	2400	2	2	2	2	2	2	2	2	2	2	2	2	2	2	2	2	3	2	2	2	2	7	2	2	2	2	2	2	2	
ENAIOESO01	856501	859500	3000	2	2	2	2	2	2	2	2	2	2	2	2	2	2	2	4	2	2	2	4	2	2	2	2	1	2	2	2	2	Cb.1.18420 (+);
ENAIOESO01	1048501	1050300	1800	2	2	2	2	2	2	2	2	2	2	2	2	2	2	2	2	2	2	2	2	2	2	2	2	1	2	2	2	2	
ENAIOESO01	1424401	1425600	1200	2	2	2	2	2	2	2	2	2	2	3	2	2	2	2	2	2	2	2	2	2	2	2	2	2	2	2	2	2	
ENAIOESO01	1488901	1489800	900	2	2	2	2	2	2	1	2	2	2	2	2	2	2	2	2	2	2	2	2	1	2	2	2	2	2	2	2	2	
ENAIOESO01	1879501	1881600	2100	2	2	2	2	2	2	2	2	2	2	2	2	2	2	2	2	2	2	2	2	2	2	2	2	5	2	2	2	2	Cb.1.16320 (+);
ENAIOESO01	1917301	1919400	2100	2	2	2	2	2	2	2	2	2	2	5	2	2	2	2	2	2	2	2	2	2	2	2	2	2	2	2	2	2	
ENAIOESO01	2008801	2010900	2100	2	2	2	2	2	2	4	2	2	2	4	2	2	2	2	2	4	2	1	2	2	2	2	2	2	2	2	2	2	
ENAIOESO01	2039101	2040000	900	2	2	2	2	2	2	2	2	2	2	2	2	2	2	2	2	2	2	2	2	2	2	2	2	2	2	2	0	0	
ENAIOESO01	2124601	2132100	7500	0	2	2	2	2	2	0	2	0	2	2	1	2	0	2	1	1	1	0	2	2	2	2	2	2	4	2	5	3	
ENAIOESO01	2214901	2223900	9000	2	2	2	2	2	2	2	2	2	2	2	3	2	2	2	2	2	2	3	2	2	2	2	1	1	2	2	3	1	Cb.1.20400 (+);
ENAIOESO01	2283601	2285700	2100	2	2	2	2	2	2	2	2	2	2	2	2	2	2	2	2	2	2	2	2	2	2	2	5	3	3	2	2	2	Cb.1.16810 (+);
ENAIOESO01	2375401	2376300	900	2	2	2	2	2	2	2	2	2	2	2	2	2	2	2	2	2	2	2	2	2	2	2	2	2	2	2	2	1	
ENAIOESO01	2550901	2551800	900	2	2	2	2	2	2	2	2	2	2	2	2	2	2	2	2	2	2	2	2	2	2	2	2	2	6	2	2	2	
ENAIOESO01	2556601	2557500	900	2	2	2	2	2	2	2	2	2	2	2	2	2	2	2	2	2	2	2	2	2	2	2	4	4	3	2	2	2	
ENAIOESO01	158401	184800	26400	3	2	2	2	2	2	2	2	2	2	2	2	2	2	2	2	2	2	2	2	2	2	2	2	2	3	2	2	2	Cb.1.41010 (+); Cb.1.41080 (+); Cb.1.41100 (+); Cb.1.40790 (+); Cb.1.41020 (+); Cb.1.41060 (+); Cb.1.41090 (+); Cb.1.41110 (+); Cb.1.40830 (+); Cb.1.41000 (+);
ENAIOESO01	199801	201900	2100	2	2	3	3	2	7	2	2	2	2	2	2	2	2	2	2	2	2	2	2	2	7	6	2	2	6	7	2	2	
ENAIOESO01	29401	34200	4800	2	2	2	2	2	2	2	2	2	2	2	2	2	1	2	2	2	2	2	2	2	2	2	1	1	2	2	2	1	Cb.1.63780 (-); Cb.1.62470 (-);
ENAIOESO01	53701	54600	900	2	2	2	2	2	2	2	2	2	2	2	2	2	2	2	2	2	2	2	2	2	2	2	2	2	2	2	2	1	
ENAIOESO01	229201	230100	900	2	2	2	2	2	1	2	2	2	2	2	2	2	2	2	2	2	2	2	2	2	1	2	2	2	1	2	2	1	
ENAIOESO01	304501	305400	900	2	2	2	2	2	2	2	2	2	2	2	2	2	2	2	2	2	2	2	2	2	2	2	2	2	2	2	2	1	
ENAIOESO01	469501	475500	6000	2	2	2	2	2	2	3	2	2	2	2	2	2	2	2	2	2	2	2	2	2	3	2	2	2	2	2	3	3	Cb.1.63890 (-); Cb.1.64000 (-); Cb.1.62320 (-);
ENAIOESO01	597601	598500	900	2	2	2	2	2	2	2	2	2	2	2	2	2	2	2	2	2	2	2	2	2	2	2	2	2	2	2	1	2	Cb.1.61120 (+);
ENAIOESO01	861601	863400	1800	2	2	2	2	2	2	2	2	2	2	2	2	2	2	2	2	2	2	2	2	2	2	2	2	2	2	2	2	3	
ENAIOESO01	884401	886800	2400	2	2	2	2	2	5	2	3	2	3	2	2	3	2	3	3	2	3	2	3	3	1	1	1	1	1	1	3	3	
ENAIOESO01	981901	983400	1500	2	2	2	2	2	2	2	2	2	2	2	2	2	2	2	2	2	2	2	2	2	2	2	2	2	2	2	2	1	
ENAIOESO01	986701	987900	1200	2	2	2	1	2	2	2	2	2	2	2	2	2	2	2	2	2	2	2	2	2	2	2	2	2	2	2	1	1	
ENAIOESO01	1078501	1080000	1500	2	2	2	2	2	2	2	2	2	2	2	2	2	3	2	2	2	2	2	2	2	2	2	2	2	2	2	2	2	Cb.1.63680 (-);
ENAIOESO01	1087501	1088400	900	2	2	2	2	2	2	2	2	2	2	2	2	2	2	2	2	2	2	2	2	2	2	2	2	2	2	2	2	1	
ENAIOESO01	1111201	1112700	1500	2	2	2	2	2	2	2	2	2	2	2	2	2	2	2	2	2	2	2	2	2	2	2	2	2	2	2	2	1	
ENAIOESO01	1185001	1189800	4800	2	2	2	2	2	2	2	2	2	2	2	2	2	2	2	2	2	2	2	2	2	2	2	0	0	2	2	2	2	Cb.1.60850 (+); Cb.1.61260 (+);
ENAIOESO01	1249501	1251300	1800	2	2	2	2	2	2	2	2	2	2	2	2	2	3	2	2	2	2	2	2	2	2	2	2	2	2	2	2	2	Cb.1.61070 (+);
ENAIOESO01	1410601	1415700	5100	2	2	3	3	2	2	2	2	2	2	2	2	2	2	2	2	2	2	2	2	2	2	2	2	2	2	2	2	2	
ENAIOESO01	1	9900	9900	2	2	2	2	2	2	2	2	2	2	2	2	2	2	2	2	2	2	2	2	2	2	2	1	1	2	1	2	2	
ENAIOESO01	156601	157500	900	2	2	2	2	2	2	2	2	3	2	2	2	2	2	2	2	2	2	2	2	2	2	2	2	2	2	2	2	2	
ENAIOESO01	163501	165600	2100	2	2	2	2	2	2	2	2	2	2	2	2	2	2	3	2	2	2	2	2	2	2	2	2	2	2	2	2	2	
ENAIOESO01	173401	177000	3600	2	2	2	2	2	2	3	2	2	2	2	3	2	2	2	2	2	2	3	3	2	2	2	2	2	2	2	2	2	
ENAIOESO01	177901	181200	3300	2	2	2	2	2	2	3	2	2	2	2	3	2	2	2	2	2	2	3	3	2	2	2	2	2	2	2	2	2	Cb.1.47040 (+); Cb.1.47130 (+);
ENAIOESO01	185701	193625	7925	2	2	2	2	2	2	3	2	2	2	2	3	2	2	2	2	2	2	3	2	2	2	2	2	2	2	2	2	2	Cb.1.47210 (+);
ENAIOESO01	272401	274800	2400	2	2	2	2	2	2	2	2	2	2	2	2	2	2	2	2	2	2	2	2	2	2	3	3	3	2	2	2	2	
ENAIOESO01	346201	347400	1200	2	2	2	2	2	2	2	2	3	3	2	2	2	2	2	2	3	2	2	2	2	2	2	2	2	2	2	2	2	Cb.1.72760 (-);
ENAIOESO01	357601	359100	1500	2	2	2	2	2	2	2	2	2	2	2	2	2	2	2	2	2	2	2	1	2	2	2	1	1	1	2	2	2	Cb.1.71980 (-); Cb.1.74250 (-);
ENAIOESO01	418801	420600	1800	2	3	2	2	2	2	2	2	2	2	2	2	2	2	2	2	2	2	2	2	2	2	2	2	2	2	2	2	2	Cb.1.72830 (-);
ENAIOESO01	497701	508800	11100	3	1	3	3	3	2	3	2	5	1	2	2	2	2	2	2	2	3	2	3	2	2	3	3	3	2	3	2	5	Cb.1.73760 (-); Cb.1.73860 (-); Cb.1.74650 (-); Cb.1.74710 (-); Cb.1.74970 (-); Cb.1.74980 (-); Cb.1.74990 (-); Cb.1.74530 (-);
ENAIOESO01	514501	515700	1200	2	2	2	2	3	2	2	2	2	2	2	2	2	2	2	2	2	2	2	2	2	2	2	2	2	2	2	2	2	Cb.1.73100 (-);
ENAIOESO01	708901	711300	2400	2	2	2	2	2	2	2	2	0	2	2	2	2	2	2	2	2	2	2	2	2	1	1	0	1	1	1	2	2	
ENAIOESO01	713401	717300	3900	2	2	3	3	2	2	2	2	5	2	2	2	2	2	2	2	2	2	2	2	2	4	2	4	4	2	4	2	2	Cb.1.72390 (-);
ENAIOESO01	729301	730200	900	2	2	1	1	2	2	2	2	2	2	2	2	2	2	2	2	2	2	2	2	2	2	2	2	2	2	2	2	2	
ENAIOESO01	818101	819600	1500	2	2	4	4	2	2	2	2	4	2	2	2	2	2	2	2	2	2	2	2	2	5	2	2	2	4	4	2	2	
ENAIOESO01	823501	825300	1800	2	2	1	1	2	2	2	2	1	2	2	2	2	2	2	2	2	2	2	2	2	0	2	1	1	0	0	2	0	Cb.1.74860 (-);
ENAIOESO01	936301	938400	2100	2	2	4	5	2	2	2	2	3	2	2	2	2	2	2	2	2	2	2	2	2	4	2	2	2	4	2	2	3	Cb.1.74900 (-);
ENAIOESO01	942301	978300	36000	2	2	3	2	2	2	2	2	3	2	2	2	2	2	2	2	2	2	2	2	2	3	2	2	2	3	2	2	3	Cb.1.73480 (-); Cb.1.73770 (-); Cb.1.74180 (-); Cb.1.74790 (-); Cb.1.73570 (-); Cb.1.74910 (-); Cb.1.74780 (-);
ENAIOESO01	980701	991500	10800	2	2	4	4	2	2	2	2	1	2	2	2	2	2	2	2	2	2	2	2	2	3	2	2	2	4	2	2	4	Cb.1.74920 (-);
ENAIOESO01	991801	997500	5700	2	2	2	2	2	2	2	2	1	2	2	2	2	2	2	2	2	2	2	2	2	2	2	2	2	2	2	2	2	
ENAIOESO01	1007101	1009500	2400	2	2	5	6	2	2	2	2	2	2	2	2	2	2	2	2	2	2	2	2	2	6	2	2	2	6	2	2	6	Cb.1.75020 (-);
ENAIOESO01	1069501	1070700	1200	2	2	2	2	2	2	2	2	2	2	2	2	2	2	2	2	2	2	2	3	2	2	2	2	2	2	2	2	2	Cb.1.73720 (-); Cb.1.74140 (-);
ENAIOESO01	1101601	1103100	1500	2	2	2	2	2	2	2	2	2	2	2	2	2	2	2	2	2	2	2	2	2	3	2	2	2	2	2	2	2	
ENAIOESO01	1104601	1106400	1800	2	2	2	2	2	2	2	2	2	2	2	3	2	2	2	2	2	3	2	2	2	2	2	2	2	2	2	2	2	
ENAIOESO01	1200001	1210200	10200	2	2	2	2	2	2	2	2	2	2	2	2	2	2	2	2	2	2	2	2	2	2	2	2	2	3	2	2	2	Cb.1.71920 (-); Cb.1.72820 (-);
ENAIOESO01	1211401	1220400	9000	2	2	2	2	2	2	2	2	2	2	2	2	2	2	2	2	2	2	2	2	2	2	2	2	2	3	2	2	2	Cb.1.73890 (-); Cb.1.72380 (-);
ENAIOESO01	1223401	1224600	1200	2	2	2	2	2	2	2	2	2	2	2	2	2	2	2	2	2	2	2	2	2	2	2	2	2	3	2	2	2	
ENAIOESO01	1240201	1242000	1800	2	2	2	2	2	2	2	2	2	2	2	2	2	2	2	2	2	2	2	2	2	0	2	2	2	0	2	2	2	
ENAIOESO01	1303801	1305000	1200	2	2	2	2	2	2	2	2	2	2	2	2	2	2	2	2	2	1	2	2	2	2	2	2	2	2	2	2	2	Cb.1.73070 (-);
ENAIOESO01	1406101	1407300	1200	2	2	2	2	2	2	2	2	2	2	2	2	2	2	2	2	2	2	2	2	2	2	2	2	2	2	2	2	1	
ENAIOESO01	130801	132000	1200	2	2	2	2	2	2	2	2	2	2	2	2	2	2	2	2	2	2	2	2	2	2	2	2	2	2	2	1	2	
ENAIOESO01	145801	148800	3000	2	2	2	2	2	2	2	3	3	2	2	2	2	2	2	2	2	2	2	2	2	2	4	4	4	2	4	3	2	Cb.1.56300 (-); Cb.1.56570 (-);
ENAIOESO01	308401	309300	900	2	2	1	1	2	2	2	2	2	2	2	2	2	2	2	2	2	2	2	2	2	1	2	2	2	1	2	2	2	Cb.1.55910 (+);
ENAIOESO01	364501	365400	900	2	2	2	2	2	2	2	2	2	2	2	2	2	2	2	2	2	2	2	2	2	2	2	2	2	2	2	1	2	
ENAIOESO01	3601	4800	1200	2	2	2	2	2	2	2	2	2	2	2	2	2	2	2	2	2	2	2	2	2	2	2	2	2	3	2	2	2	Cb.1.33290 (-);
ENAIOESO01	63901	66300	2400	2	2	2	2	2	2	2	2	2	2	2	2	2	2	3	2	2	2	2	2	2	1	0	0	0	0	0	2	2	
ENAIOESO01	175501	176400	900	2	2	2	2	2	2	2	2	2	2	2	2	2	2	2	2	2	2	2	2	2	2	2	2	2	2	2	2	1	
ENAIOESO01	28801	30900	2100	4	2	2	2	4	2	2	2	3	2	2	3	2	2	2	2	2	3	2	3	2	2	2	2	2	2	2	2	2	Cb.1.30070 (-); Cb.1.30330 (-);
ENAIOESO01	70501	71400	900	2	2	2	2	2	2	2	2	2	2	2	2	2	2	2	2	2	2	2	2	2	2	2	2	1	2	2	2	2	
ENAIOESO01	182701	183600	900	2	2	2	2	2	2	2	2	2	2	2	2	2	2	2	2	2	2	2	2	2	2	2	2	2	2	2	1	2	
ENAIOESO01	187201	188400	1200	2	2	2	2	2	2	2	2	2	2	2	2	2	2	2	2	2	2	2	2	2	2	2	2	2	2	2	1	1	
ENAIOESO01	43801	44700	900	2	2	2	2	2	2	2	2	2	2	2	2	2	2	2	2	2	2	2	2	2	2	2	0	0	2	2	2	2	Cb.1.54470 (-); Cb.1.54650 (-);
ENAIOESO01	79201	94200	15000	2	2	2	2	2	2	2	2	2	2	2	2	2	2	2	2	2	2	2	2	2	2	2	1	1	2	2	2	2	Cb.1.54480 (-); Cb.1.54860 (-); Cb.1.54880 (-); Cb.1.54790 (-);
ENAIOESO01	114601	123900	9300	2	2	2	2	2	2	2	2	2	2	2	2	2	2	2	2	2	2	2	2	2	5	1	1	1	5	1	2	2	Cb.1.54490 (-); Cb.1.54530 (-); Cb.1.54710 (-);
ENAIOESO01	199201	200400	1200	2	2	2	2	2	2	2	2	2	2	2	2	2	2	2	2	2	2	2	2	2	2	2	2	2	2	2	2	1	
ENAIOESO01	309601	310500	900	2	2	2	2	2	2	2	2	2	2	2	2	2	2	2	2	2	2	2	2	2	2	2	4	4	2	2	2	2	Cb.1.54450 (+);
ENAIOESO01	312601	315600	3000	2	2	2	2	2	2	2	2	2	2	2	2	2	2	2	2	2	2	2	2	2	2	2	3	3	2	2	2	2	
ENAIOESO01	323401	324600	1200	2	2	2	2	2	2	2	2	2	2	2	2	2	2	2	2	2	2	2	2	2	2	2	3	3	2	2	2	2	Cb.1.54420 (+);
ENAIOESO01	78901	79800	900	2	2	2	2	2	2	2	2	2	2	2	2	2	2	2	2	3	2	3	2	2	2	2	2	2	2	2	2	2	Cb.1.53590 (-); Cb.1.54150 (-);
ENAIOESO01	95401	97800	2400	2	2	2	2	2	2	2	2	2	2	3	2	2	2	2	2	2	3	2	2	2	2	2	2	2	2	2	2	2	Cb.1.54160 (-); Cb.1.54220 (-); Cb.1.54230 (-);
ENAIOESO01	153301	154200	900	2	2	2	2	2	2	2	2	2	2	2	2	2	2	2	3	2	2	2	2	2	2	2	2	2	2	2	2	2	Cb.1.53790 (-);
ENAIOESO01	38101	39000	900	2	2	2	2	2	2	2	2	2	2	2	2	2	2	2	2	2	2	2	2	2	2	2	2	2	2	2	2	1	
ENAIOESO01	111601	112500	900	2	2	2	2	2	2	2	2	2	2	2	2	2	2	2	2	2	2	2	2	2	2	2	2	2	2	4	2	2	
ENAIOESO01	191401	192300	900	2	4	2	2	2	2	2	2	2	4	2	2	2	2	2	2	2	4	2	4	2	2	2	1	1	2	2	2	2	
ENAIOESO01	262201	263700	1500	2	2	2	2	3	2	2	2	6	2	2	2	2	2	2	2	2	2	2	2	2	6	2	2	2	6	2	2	3	Cb.1.60020 (+);
ENAIOESO01	34801	35700	900	2	2	2	2	2	2	2	2	2	2	2	2	2	2	2	2	2	2	2	2	2	2	2	0	0	2	2	2	2	
ENAIOESO01	833701	836100	2400	2	1	2	2	2	2	2	2	2	2	2	2	2	2	2	2	2	2	2	2	3	2	2	2	2	2	2	2	2	
ENAIOESO01	1010101	1011000	900	2	2	2	2	2	2	3	2	2	2	2	3	3	2	2	3	3	3	2	2	2	2	2	2	2	2	2	2	2	
ENAIOESO01	113401	114300	900	2	2	2	2	2	2	2	2	2	2	2	2	2	2	2	2	2	2	2	2	2	2	1	1	1	2	2	2	2	
ENAIOESO01	482101	484500	2400	2	2	2	2	2	2	2	2	2	3	2	2	3	3	3	3	2	4	2	2	4	2	2	3	2	2	2	4	2	Cb.1.53270 (-);
ENAIOESO01	70201	72300	2100	2	2	2	2	2	2	2	2	4	2	2	2	3	2	2	2	2	2	2	2	2	2	2	2	2	2	2	2	2	
ENAIOESO01	76801	92700	15900	2	2	2	2	2	2	2	2	3	2	2	2	2	2	2	2	2	2	2	2	2	2	2	2	2	2	2	2	2	Cb.1.40360 (+); Cb.1.40230 (+); Cb.1.40300 (+); Cb.1.40620 (+); Cb.1.40670 (+); Cb.1.40740 (+);
ENAIOESO01	105301	111600	6300	2	2	2	2	2	2	2	2	4	2	2	2	2	2	2	2	2	2	2	2	2	2	2	2	2	2	2	2	2	Cb.1.40150 (+);
ENAIOESO01	130801	132000	1200	2	2	2	2	2	2	2	2	1	2	2	2	2	2	2	2	2	2	2	2	2	2	2	2	2	2	2	2	2	Cb.1.40090 (+);
ENAIOESO01	261601	262500	900	2	2	2	2	2	2	2	2	2	2	2	2	2	2	2	2	2	2	2	2	2	2	2	0	0	2	1	2	2	
ENAIOESO01	1	3000	3000	2	1	3	3	2	3	2	2	3	2	2	2	2	2	1	2	2	1	2	2	2	3	3	2	2	3	3	3	3	
ENAIOESO01	10801	12300	1500	2	2	2	2	2	2	2	2	3	2	2	2	2	2	2	3	2	2	2	2	2	2	2	2	2	2	2	2	2	Cb.1.00430 (+);
ENAIOESO01	22201	23100	900	2	2	2	2	2	2	2	2	2	2	2	2	2	2	2	2	2	3	2	2	2	2	2	2	2	2	2	2	2	Cb.1.00400 (+);
ENAIOESO01	36601	38700	2100	2	2	2	2	2	3	3	2	4	3	2	2	2	2	2	2	2	2	2	2	2	4	2	2	2	2	2	3	4	Cb.1.00410 (+);
ENAIOESO01	432901	433800	900	2	2	2	1	2	1	2	2	2	2	2	2	2	2	2	2	2	2	2	2	2	2	1	2	2	1	1	2	2	
ENAIOESO01	478201	479700	1500	2	2	4	4	2	4	2	2	4	2	2	2	2	2	2	2	2	2	2	2	2	4	4	2	2	4	4	2	4	
ENAIOESO01	492001	493200	1200	2	2	4	4	2	4	2	2	2	2	2	2	2	2	2	2	2	2	2	2	2	2	2	2	2	4	5	2	2	
ENAIOESO01	498601	501000	2400	2	2	4	4	2	4	2	2	4	2	2	2	2	1	2	2	2	1	2	2	2	4	2	2	2	4	4	4	4	Cb.1.01670 (-); Cb.1.00390 (+);
ENAIOESO01	517201	521100	3900	2	2	3	3	2	2	2	2	2	2	2	2	2	2	2	2	2	2	2	2	2	4	2	2	2	2	2	2	2	Cb.1.00360 (+); Cb.1.00790 (-);
ENAIOESO01	581401	582600	1200	2	2	2	2	2	2	2	2	2	2	2	2	2	2	2	2	2	2	2	2	2	2	2	2	2	2	2	1	2	
ENAIOESO01	643201	644400	1200	2	2	2	2	2	2	2	2	2	2	2	2	2	2	2	2	2	2	2	2	2	2	2	0	0	2	1	2	2	Cb.1.00520 (-);
ENAIOESO01	709201	711000	1800	2	2	2	2	2	3	2	2	2	2	2	2	2	2	3	2	2	2	2	2	2	2	3	2	2	2	3	2	2	
ENAIOESO01	773701	774600	900	2	2	2	2	2	2	2	2	2	2	2	2	2	2	2	2	2	2	2	2	2	2	1	2	2	2	2	2	2	
ENAIOESO01	788101	789300	1200	2	2	2	2	2	2	2	2	2	2	2	2	2	2	2	2	2	2	2	2	2	2	2	2	2	2	2	1	2	
ENAIOESO01	804601	806100	1500	2	2	2	2	2	2	2	2	2	2	2	2	2	2	2	2	2	2	2	2	2	2	2	2	2	2	2	1	2	
ENAIOESO01	822901	825300	2400	2	2	2	2	2	2	2	2	2	2	2	2	2	2	2	2	2	2	2	2	2	2	2	2	2	2	2	1	2	
ENAIOESO01	146401	150900	4500	2	2	2	2	2	2	2	2	3	2	2	2	2	2	2	2	2	1	2	2	2	2	1	2	2	3	1	2	2	
ENAIOESO01	162001	162900	900	2	2	6	6	2	6	2	2	6	2	2	2	2	2	2	2	2	2	2	2	2	6	2	2	2	6	2	6	6	
ENAIOESO01	400501	401400	900	2	2	2	2	2	2	2	2	2	2	1	3	1	2	2	1	2	2	2	1	2	2	2	2	2	2	2	2	2	
ENAIOESO01	598501	599400	900	2	2	2	2	2	2	2	2	2	3	2	2	2	2	2	2	2	2	2	2	2	2	2	2	2	2	2	2	2	
ENAIOESO01	1008601	1009800	1200	2	2	2	2	2	2	2	2	2	2	2	2	2	2	2	2	2	2	2	2	2	2	2	2	2	2	2	1	2	
ENAIOESO01	1056301	1058400	2100	2	2	2	2	2	2	2	2	2	2	2	2	2	2	2	2	2	2	2	3	2	2	2	2	2	2	2	2	2	
ENAIOESO01	1129501	1130400	900	2	2	1	2	2	2	2	2	1	2	2	2	2	2	2	2	2	2	2	2	2	2	2	2	2	1	2	2	1	
ENAIOESO01	1207201	1208100	900	2	2	2	2	2	2	2	2	2	2	2	2	2	2	2	2	2	2	2	2	2	2	2	2	2	2	2	2	1	
ENAIOESO01	1532701	1533900	1200	2	2	7	2	2	2	2	2	7	2	2	2	2	2	2	2	2	2	2	2	2	2	2	2	2	7	2	2	4	
ENAIOESO01	1550101	1554600	4500	2	2	6	6	2	4	2	2	6	1	2	2	1	2	2	2	2	2	1	2	2	2	2	2	2	7	2	2	6	
ENAIOESO01	1972201	1973100	900	2	2	3	3	2	2	2	2	2	2	2	2	2	2	2	2	2	2	2	2	2	2	2	2	2	2	2	2	2	
ENAIOESO01	290401	291300	900	2	2	2	2	2	2	2	2	1	2	2	2	2	2	2	2	2	2	2	2	2	2	2	2	2	2	2	2	2	
ENAIOESO01	292801	294900	2100	2	2	3	3	2	3	2	2	2	2	2	2	2	2	2	2	2	2	2	2	2	2	3	3	2	2	2	2	2	Cb.1.46370 (+);
ENAIOESO01	432301	433200	900	2	2	2	2	2	2	2	2	2	2	2	2	2	2	2	2	2	2	2	2	2	2	2	2	2	4	2	2	2	Cb.1.46320 (+);
ENAIOESO01	53401	54300	900	2	2	2	2	2	2	2	2	1	2	2	2	2	2	2	2	2	2	2	2	2	2	2	2	2	2	2	1	2	
ENAIOESO01	70501	71400	900	2	2	2	3	2	2	2	2	3	2	2	2	2	2	2	2	2	2	2	2	2	2	2	2	2	2	2	2	2	Cb.1.76610 (-);
ENAIOESO01	38101	39000	900	2	2	2	2	2	2	2	2	2	2	2	2	2	2	2	2	2	2	2	2	2	2	2	2	2	2	2	1	2	
ENAIOESO01	86701	87600	900	2	2	2	2	2	2	2	2	2	2	2	2	2	2	2	2	2	2	2	2	2	2	2	2	2	2	2	1	2	
ENAIOESO01	149101	150600	1500	2	2	2	2	2	2	2	2	2	2	2	3	2	2	2	2	2	2	2	2	2	2	2	2	2	2	2	2	2	Cb.1.37900 (+); Cb.1.38180 (+);
ENAIOESO01	167401	169500	2100	2	2	2	2	2	2	2	2	2	2	2	2	4	2	2	4	2	4	2	2	2	2	2	2	2	4	2	2	2	Cb.1.38220 (+);
ENAIOESO01	282601	283500	900	2	2	2	2	2	2	2	2	2	2	2	2	2	2	2	2	2	2	2	2	2	2	2	2	2	2	2	1	2	
ENAIOESO01	351601	352500	900	2	2	2	2	2	2	2	2	2	3	2	2	2	2	2	2	2	2	2	2	2	2	2	2	2	2	2	2	2	
ENAIOESO01	489601	490500	900	2	1	2	2	2	2	2	2	2	2	2	2	2	2	2	2	2	2	2	2	2	2	2	2	2	2	2	2	2	Cb.1.38030 (+);
ENAIOESO01	523801	525300	1500	2	2	0	0	2	1	2	2	0	2	2	2	2	2	2	2	2	2	2	2	2	0	0	1	1	0	0	0	1	
ENAIOESO01	676801	678000	1200	2	2	4	4	1	2	2	2	2	2	2	2	4	4	1	2	2	2	2	2	2	2	2	2	2	2	2	2	2	
ENAIOESO01	18601	19800	1200	2	2	2	2	2	2	2	2	2	2	2	2	2	2	2	2	2	2	2	2	2	2	1	2	2	2	2	2	2	
ENAIOESO01	31801	35100	3300	2	2	2	2	2	2	2	2	2	2	2	2	2	2	3	2	2	2	2	2	2	2	2	2	2	2	2	2	2	
ENAIOESO01	269401	270300	900	2	2	2	2	2	2	2	2	2	2	2	2	2	2	2	2	2	2	2	2	2	2	2	2	2	2	2	2	1	
ENAIOESO01	295501	296700	1200	2	2	2	2	2	2	2	2	2	2	2	2	2	2	2	2	2	2	2	2	2	2	1	2	2	2	2	1	1	
ENAIOESO01	1	2400	2400	2	2	2	2	2	2	2	2	2	2	2	2	2	2	2	2	2	2	2	2	2	2	1	2	2	2	2	2	2	
ENAIOESO01	220501	221400	900	2	2	2	2	2	2	2	2	2	2	2	2	2	2	2	2	2	2	2	2	2	2	2	2	2	2	2	1	2	
ENAIOESO01	233701	235500	1800	2	2	2	2	2	2	2	2	2	2	2	2	2	2	2	2	2	2	2	2	3	2	2	3	2	2	2	2	2	Cb.1.70120 (+);
ENAIOESO01	308401	309300	900	1	2	2	2	1	2	2	2	2	2	2	2	2	2	2	2	2	2	2	2	2	2	2	2	2	2	2	2	2	
ENAIOESO01	314701	315600	900	2	2	2	2	2	2	2	2	2	2	2	2	2	2	2	2	2	2	2	2	2	2	2	2	2	2	2	0	2	
ENAIOESO01	316201	317400	1200	3	2	2	2	2	2	2	4	2	2	4	4	2	2	2	2	2	2	2	4	2	2	2	2	2	2	2	2	2	
ENAIOESO01	350701	352200	1500	2	2	2	2	2	2	2	2	2	2	2	2	2	2	2	2	2	2	2	2	2	2	1	2	2	2	2	1	1	
ENAIOESO01	417001	420600	3600	2	2	2	2	2	2	2	2	2	2	2	2	2	2	2	2	2	2	2	2	2	2	2	1	0	2	2	2	2	Cb.1.69220 (+);
ENAIOESO01	440401	459300	18900	2	2	3	3	2	2	2	2	3	2	2	2	2	2	2	2	2	2	2	2	2	3	1	1	1	3	1	3	3	Cb.1.69240 (+); Cb.1.69250 (+); Cb.1.69550 (+); Cb.1.69360 (+); Cb.1.69570 (+);
ENAIOESO01	1	1500	1500	2	2	2	2	2	2	2	2	2	2	2	2	2	2	2	2	2	2	2	2	2	2	2	1	1	2	2	2	2	
ENAIOESO01	149401	150300	900	2	2	2	2	2	2	2	2	2	2	2	2	2	2	2	2	2	2	2	2	2	2	2	2	2	2	2	1	2	
ENAIOESO01	155701	156600	900	2	2	2	1	2	2	2	2	2	2	2	2	2	2	2	2	2	2	2	2	2	2	2	2	2	2	2	2	2	
ENAIOESO01	180301	181200	900	2	2	2	2	2	2	2	2	2	2	2	2	2	2	2	2	2	2	2	2	2	2	2	2	2	2	2	1	2	
ENAIOESO01	297601	298500	900	2	2	2	2	2	2	2	2	2	2	2	2	2	2	2	3	2	2	2	2	2	2	2	2	2	2	2	2	2	Cb.1.58670 (-);
ENAIOESO01	341701	342900	1200	2	2	2	2	2	2	2	2	2	2	2	2	2	2	2	2	2	2	2	2	2	2	2	1	0	2	2	2	2	
ENAIOESO01	196801	202500	5700	2	2	2	2	2	2	2	2	2	2	3	2	2	2	2	2	2	2	2	2	2	2	2	2	2	2	2	2	2	Cb.1.49260 (-); Cb.1.49680 (-); Cb.1.49340 (-);
ENAIOESO01	210901	212100	1200	2	2	2	2	2	2	2	2	2	2	2	2	2	2	2	2	2	2	2	2	2	2	2	2	2	2	2	2	1	
ENAIOESO01	13801	14700	900	2	2	2	2	2	2	2	2	2	2	2	2	2	2	2	2	2	2	2	2	2	2	2	2	2	1	2	2	2	
ENAIOESO01	117901	120600	2700	2	2	2	2	1	2	2	3	2	2	2	2	3	2	2	2	2	2	2	2	2	2	2	2	2	2	2	2	2	Cb.1.75330 (-);
ENAIOESO01	124501	125400	900	2	2	2	2	2	2	2	2	2	2	2	2	2	2	2	2	2	2	2	2	2	2	2	2	2	2	2	1	2	
ENAIOESO01	1	1500	1500	2	2	2	2	2	4	2	2	3	2	2	2	2	2	2	2	2	2	2	2	2	4	4	4	2	2	4	2	2	
ENAIOESO01	11101	12000	900	2	2	2	3	2	2	2	2	2	2	2	2	2	2	2	2	2	2	2	2	2	2	2	2	2	2	2	4	4	
ENAIOESO01	108001	108900	900	2	2	2	1	2	2	2	2	2	2	2	2	2	2	2	2	2	2	2	2	2	2	2	2	2	2	2	2	2	
ENAIOESO01	179401	180300	900	2	2	2	2	2	2	2	2	2	2	2	2	2	2	2	2	2	2	2	2	2	2	2	2	2	2	2	2	1	
ENAIOESO01	219901	221100	1200	2	2	2	2	2	2	2	2	2	2	2	2	2	2	2	2	2	2	2	2	2	2	2	2	2	2	2	2	1	
ENAIOESO01	237601	238800	1200	2	2	2	1	2	2	2	2	2	2	2	2	2	2	2	2	2	2	2	2	2	2	2	2	2	2	2	2	1	
ENAIOESO01	265201	267000	1800	3	2	2	2	2	2	2	2	2	2	2	2	2	2	2	2	2	2	2	2	2	2	2	2	2	2	2	2	2	Cb.1.30810 (+);
ENAIOESO01	529501	532500	3000	2	2	2	2	2	2	2	2	2	2	2	2	2	2	2	2	2	2	2	2	2	2	2	1	2	2	2	2	2	Cb.1.31610 (+);
ENAIOESO01	547501	548400	900	2	2	2	2	2	2	2	2	2	2	2	2	2	2	2	2	2	2	2	2	2	2	2	0	2	2	2	2	2	
ENAIOESO01	561301	562200	900	2	2	2	2	2	2	2	2	2	2	2	2	2	2	2	2	2	2	2	2	2	2	1	1	2	2	1	2	2	
ENAIOESO01	566101	567000	900	2	2	2	2	2	2	2	2	2	2	2	2	2	2	2	2	2	2	2	2	2	2	2	2	2	2	1	2	2	Cb.1.32200 (+);
ENAIOESO01	15601	18600	3000	2	2	2	2	2	2	2	2	2	2	2	2	2	2	2	2	2	2	2	2	2	2	3	2	2	2	2	2	2	Cb.1.57260 (+);
ENAIOESO01	33901	35100	1200	2	2	2	2	2	2	2	2	2	2	2	2	2	2	2	2	2	2	2	2	2	2	2	2	2	2	2	2	1	Cb.1.57500 (+);
ENAIOESO01	164401	165900	1500	2	2	1	2	2	1	2	2	1	2	2	2	2	2	2	2	2	2	2	2	2	2	1	2	2	2	2	1	1	
ENAIOESO01	166501	169200	2700	2	2	2	2	2	2	2	3	2	2	2	2	2	2	2	2	2	2	2	2	2	2	2	2	2	2	2	2	2	Cb.1.57540 (+); Cb.1.57760 (+);
ENAIOESO01	184501	186454	1954	2	2	2	2	2	2	2	2	3	2	2	2	2	2	2	2	2	2	2	2	2	2	2	2	2	2	2	2	4	
ENAIOESO01	448501	449400	900	2	2	2	4	2	2	2	2	2	2	2	2	2	2	2	2	2	2	2	2	2	2	2	2	2	2	2	2	2	Cb.1.51380 (-);
ENAIOESO01	477301	482100	4800	3	2	2	2	4	4	4	2	3	4	4	2	2	2	4	4	1	4	2	2	4	2	2	2	2	2	2	4	2	Cb.1.51930 (-); Cb.1.52350 (-);
ENAIOESO01	35401	36900	1500	2	2	2	2	2	2	2	2	2	2	2	2	2	2	2	2	2	2	2	2	2	2	2	2	2	2	2	1	1	
ENAIOESO01	120301	121500	1200	2	2	2	2	2	2	2	2	2	2	2	2	2	2	2	2	2	2	2	2	2	2	2	2	2	2	2	1	2	
ENAIOESO01	130201	131100	900	2	2	2	2	2	1	2	2	2	2	2	2	2	2	2	2	1	2	2	2	2	2	2	0	0	2	1	2	2	Cb.1.68360 (+);
ENAIOESO01	140101	144300	4200	2	2	2	2	2	2	2	2	2	2	2	2	2	2	2	2	2	2	2	2	2	2	2	2	2	3	2	2	2	Cb.1.68020 (+); Cb.1.68050 (+);
ENAIOESO01	117001	120000	3000	2	2	3	3	3	1	3	2	0	3	3	3	2	2	2	2	2	2	2	2	2	3	0	0	0	2	0	2	3	Cb.1.36530 (+); Cb.1.37320 (+); Cb.1.37330 (+);
ENAIOESO01	398101	399000	900	2	2	2	2	2	2	2	2	2	2	2	2	2	2	2	2	2	2	2	2	2	2	2	2	0	2	2	2	2	Cb.1.36670 (+);
ENAIOESO01	410401	411300	900	2	2	2	2	2	2	2	2	2	2	2	2	2	2	2	2	2	2	2	2	2	7	2	2	2	2	2	2	2	Cb.1.35660 (+);
ENAIOESO01	422401	426900	4500	2	2	2	2	2	5	2	2	5	2	2	2	2	2	2	2	2	2	2	2	2	5	3	2	3	5	3	2	2	Cb.1.36210 (+);
ENAIOESO01	454201	457500	3300	2	2	2	2	2	2	2	2	2	2	2	2	2	2	2	2	2	2	2	2	2	3	2	1	1	3	2	2	2	Cb.1.35510 (+);
ENAIOESO01	465301	466200	900	2	2	2	2	2	2	2	2	2	2	2	2	1	2	2	2	2	2	2	2	2	1	2	2	2	2	2	2	2	
ENAIOESO01	538801	540000	1200	2	2	2	2	2	2	2	2	2	2	2	2	1	2	1	2	3	2	2	2	2	2	2	2	2	2	2	2	2	
ENAIOESO01	607501	608400	900	2	2	2	2	2	2	2	2	2	2	2	2	2	2	2	2	2	2	2	2	2	2	2	1	1	2	2	2	2	
ENAIOESO01	617101	618000	900	2	2	2	2	2	2	2	2	2	2	2	2	2	2	2	2	2	2	2	2	2	2	1	2	2	2	2	2	2	
ENAIOESO01	660901	661800	900	2	2	2	2	2	2	2	2	2	2	2	2	2	2	2	2	2	2	2	2	2	2	2	2	2	1	2	2	2	
ENAIOESO01	1	2400	2400	2	2	3	3	2	2	2	2	2	2	2	2	2	2	2	2	2	2	2	2	2	2	2	1	0	2	2	3	3	
ENAIOESO01	11701	12600	900	2	2	2	2	2	2	2	2	2	2	2	2	2	2	2	2	2	2	2	2	2	2	2	2	4	2	2	2	2	
ENAIOESO01	13201	16500	3300	2	2	2	2	2	2	2	2	2	2	2	2	2	2	2	2	2	2	2	2	2	3	2	2	2	3	2	2	2	Cb.1.58410 (-);
ENAIOESO01	152101	153300	1200	2	2	4	4	2	2	2	2	2	2	2	2	2	2	2	2	2	2	2	2	2	4	2	2	4	4	2	4	4	Cb.1.58440 (-);
ENAIOESO01	214501	215917	1417	2	2	4	4	2	2	2	2	2	2	2	2	2	2	2	2	2	2	2	2	2	5	2	2	2	4	2	4	4	
ENAIOESO01	186001	186900	900	2	2	2	2	2	2	2	2	2	2	2	2	2	2	2	2	2	2	2	2	2	2	2	2	2	1	2	2	2	
ENAIOESO01	414301	415500	1200	2	2	1	1	2	2	2	2	2	2	2	2	2	2	2	2	2	2	2	2	2	2	2	2	2	1	1	2	1	
ENAIOESO01	531601	532500	900	2	2	2	2	2	2	2	2	1	2	2	2	2	2	2	2	2	2	2	2	2	2	2	2	2	2	2	2	2	Cb.1.77690 (-);
ENAIOESO01	533701	536700	3000	1	2	2	3	1	2	1	2	2	2	1	2	2	2	2	2	2	1	2	2	2	5	5	4	2	4	5	2	3	Cb.1.77660 (-);
ENAIOESO01	1	4500	4500	2	2	2	2	2	2	2	2	2	2	2	1	2	2	2	2	2	2	2	2	2	2	2	2	2	2	2	2	2	
ENAIOESO01	54301	55200	900	2	2	2	2	2	2	2	2	2	2	2	2	2	2	2	2	2	2	2	2	2	2	2	2	2	1	2	2	2	Cb.1.37410 (-);
ENAIOESO01	43801	45600	1800	2	2	2	2	2	2	2	2	2	2	2	2	2	2	2	2	2	2	2	2	2	2	2	2	2	2	2	1	1	
ENAIOESO01	125101	126000	900	2	2	2	2	2	1	2	2	2	2	2	2	2	2	2	2	2	2	2	2	2	2	2	2	2	2	2	2	2	
ENAIOESO01	185401	188100	2700	2	2	2	2	2	2	2	2	2	2	2	2	2	2	2	2	2	2	2	2	2	2	3	2	2	2	2	2	2	Cb.1.48340 (-); Cb.1.48670 (-);
ENAIOESO01	429901	431100	1200	2	2	2	2	2	2	2	2	2	2	2	1	2	2	2	2	2	2	2	2	2	2	3	2	2	2	3	3	2	
ENAIOESO01	438301	439200	900	2	2	2	2	2	2	2	2	1	2	2	2	2	2	2	2	2	2	2	2	2	2	2	2	2	2	2	1	1	
ENAIOESO01	452401	453600	1200	2	2	3	2	2	3	2	2	3	2	2	3	2	2	2	2	2	2	2	2	2	2	2	2	2	2	2	4	3	
ENAIOESO01	490801	494400	3600	2	2	3	2	2	3	2	2	3	2	1	2	2	2	2	2	2	2	2	2	3	1	1	2	2	1	1	3	3	Cb.1.48380 (-);
ENAIOESO01	565501	575700	10200	2	2	2	2	2	2	2	2	2	2	2	2	2	3	2	2	2	2	2	2	2	2	2	2	2	2	2	2	2	Cb.1.47640 (+); Cb.1.47720 (+); Cb.1.47740 (+); Cb.1.48020 (+);
ENAIOESO01	15901	17100	1200	2	2	2	2	2	3	2	2	2	2	2	2	2	2	2	2	2	2	2	2	2	2	2	2	2	2	2	2	2	Cb.1.42200 (+);
ENAIOESO01	98701	99600	900	2	2	4	5	2	4	2	2	4	2	2	2	2	2	2	2	2	2	2	2	2	5	4	4	4	5	4	5	5	Cb.1.41960 (+);
ENAIOESO01	161701	162600	900	2	2	2	2	2	2	2	2	2	2	2	2	2	3	2	4	2	2	2	2	2	2	2	2	2	2	2	2	2	
ENAIOESO01	227401	228300	900	2	2	2	2	2	2	2	2	2	2	2	2	2	2	2	2	2	2	2	2	2	2	2	2	4	4	2	2	2	
ENAIOESO01	139801	143400	3600	4	2	2	2	4	2	2	2	2	2	2	3	4	3	3	2	2	2	2	3	2	3	2	2	2	3	4	2	2	Cb.1.25680 (+); Cb.1.26790 (+);
ENAIOESO01	228901	231900	3000	2	2	2	2	2	2	2	2	2	2	2	2	2	2	2	2	1	2	2	2	2	2	3	2	2	2	3	2	2	
ENAIOESO01	275401	277500	2100	2	2	2	2	2	2	2	2	2	2	2	2	2	2	3	2	2	2	2	2	2	2	3	2	2	2	3	2	2	Cb.1.25070 (+); Cb.1.25250 (+);
ENAIOESO01	809101	810300	1200	2	2	2	2	2	2	2	4	2	2	2	2	2	3	2	2	2	2	2	2	2	2	2	2	2	2	2	2	2	Cb.1.28640 (-);
ENAIOESO01	965701	967200	1500	2	2	2	2	1	2	1	2	2	2	2	2	1	1	1	2	2	2	1	2	2	2	4	2	2	2	4	2	2	
ENAIOESO01	971401	972600	1200	1	2	2	2	1	2	1	2	2	2	1	1	1	1	1	1	1	2	1	2	1	2	5	2	2	2	5	2	2	
ENAIOESO01	1052401	1053300	900	2	2	2	2	2	2	2	2	2	2	2	2	2	2	2	2	2	2	2	2	2	2	2	2	2	2	3	2	2	
ENAIOESO01	1225501	1226400	900	2	2	2	2	2	2	2	2	2	2	2	2	2	2	2	2	2	2	2	2	2	2	1	2	2	2	2	2	2	Cb.1.25760 (+);
ENAIOESO01	1513201	1514700	1500	4	2	2	2	4	2	2	2	2	2	3	2	4	2	2	2	2	2	2	2	2	2	2	2	2	2	2	2	2	
ENAIOESO01	1534201	1537800	3600	6	2	1	1	3	2	2	2	1	3	7	6	2	2	2	6	2	2	2	6	6	1	1	1	1	2	1	2	2	Cb.1.26850 (+);
ENAIOESO01	1692301	1695900	3600	2	2	2	2	2	2	2	2	2	2	2	2	2	2	2	4	3	2	5	2	3	2	8	5	5	2	2	2	4	Cb.1.29580 (-); Cb.1.29650 (-);
ENAIOESO01	1718101	1719000	900	2	2	2	2	2	2	2	2	2	2	2	2	2	2	2	2	2	2	2	2	2	2	1	1	2	2	2	2	2	Cb.1.27290 (-);
ENAIOESO01	85201	86100	900	2	2	2	2	2	2	2	2	2	2	2	2	2	2	2	2	2	2	2	2	2	2	2	2	1	2	2	2	2	Cb.1.34340 (-);
ENAIOESO01	98101	99300	1200	2	2	2	2	2	2	2	2	2	2	2	2	2	2	2	2	2	2	2	2	2	2	2	2	2	2	2	1	1	
ENAIOESO01	105301	108000	2700	2	2	2	2	2	2	2	2	2	2	2	2	2	2	2	2	2	2	2	2	2	2	2	3	3	2	3	2	4	Cb.1.34400 (-);
ENAIOESO01	255301	258900	3600	1	2	3	2	2	3	1	2	3	2	2	2	2	2	2	2	2	2	2	2	2	3	2	2	2	2	3	4	3	
ENAIOESO01	260401	261300	900	2	2	2	2	2	2	2	2	2	2	2	2	2	2	2	2	2	2	2	2	2	2	2	2	2	2	2	2	1	
ENAIOESO01	282901	284400	1500	2	2	2	2	2	1	2	2	2	2	2	2	2	2	2	2	2	2	2	2	2	2	2	1	1	2	2	2	2	
ENAIOESO01	336001	336900	900	2	2	2	2	2	2	2	2	2	2	3	2	2	2	2	2	2	2	2	2	2	2	2	2	2	2	2	2	2	Cb.1.34700 (-);
ENAIOESO01	11701	12600	900	2	2	2	2	2	2	2	2	2	2	2	2	2	2	2	2	2	2	2	2	2	2	2	1	2	2	2	2	2	
ENAIOESO01	42001	43800	1800	2	2	2	2	2	2	2	2	2	2	2	2	2	2	2	2	2	2	2	2	2	2	1	1	1	2	2	2	2	Cb.1.11200 (-); Cb.1.12380 (-);

ENAIOESO01	49501	52800	3300	2	2	2	2	2	2	2	2	2	2	2	2	2	2	2	2	2	2	2	2	2	2	2	2	1	2	2	2	2	
ENAIOESO01	58801	62400	3600	2	2	2	2	2	2	3	2	2	2	2	2	2	2	2	2	2	2	2	3	2	2	1	0	0	2	2	2	2	
ENAIOESO01	67201	68100	900	2	2	3	2	2	2	2	2	2	2	2	2	2	2	2	2	2	2	2	2	2	3	2	2	2	3	2	2	2	Cb.1.11800 (-); Cb.1.12220 (-); Cb.1.12440 (-);
ENAIOESO01	72601	75900	3300	2	2	3	2	2	2	2	2	2	2	2	2	2	2	2	2	2	2	2	2	2	3	2	2	2	3	2	2	2	Cb.1.11970 (-);
ENAIOESO01	79801	81300	1500	2	2	2	2	2	2	2	2	2	2	2	2	2	2	2	2	2	2	2	2	2	2	2	0	0	2	1	2	2	Cb.1.11760 (-);
ENAIOESO01	86701	90000	3300	2	2	2	2	2	2	2	2	2	2	2	2	2	2	2	2	2	2	2	2	2	2	1	0	0	2	1	2	2	
ENAIOESO01	126301	128400	2100	2	1	2	2	1	2	5	2	2	2	2	2	2	2	2	3	2	2	2	2	2	4	4	4	4	4	5	2	2	
ENAIOESO01	134101	135000	900	2	2	1	2	2	1	2	2	1	2	2	2	2	2	2	2	2	2	2	2	2	1	1	2	2	2	1	2	2	
ENAIOESO01	140101	141600	1500	2	2	2	2	2	2	2	2	2	2	2	2	2	2	2	2	2	2	2	2	2	2	1	0	0	2	1	2	2	
ENAIOESO01	159301	160200	900	2	2	2	2	2	2	2	2	2	2	2	2	2	2	2	2	2	2	2	2	2	2	1	2	1	2	2	2	2	
ENAIOESO01	196201	197100	900	2	2	1	2	2	1	2	2	1	2	2	2	2	2	2	2	2	2	2	2	2	2	2	2	2	2	2	2	2	
ENAIOESO01	233701	238500	4800	2	2	2	2	2	2	2	2	2	2	2	2	2	2	2	2	2	2	2	2	2	2	2	1	1	2	2	2	2	Cb.1.11620 (-); Cb.1.11700 (-);
ENAIOESO01	309001	309900	900	2	2	2	2	2	2	2	2	2	2	2	3	2	2	2	2	2	2	2	3	2	2	2	2	2	2	2	2	2	
ENAIOESO01	406501	407400	900	2	2	2	2	2	2	2	2	2	2	2	2	2	2	2	2	2	2	2	2	2	2	1	2	2	2	2	2	2	
ENAIOESO01	603001	603900	900	2	2	2	2	2	2	2	2	4	2	2	2	2	2	2	2	2	2	2	2	2	2	4	2	2	2	2	2	2	Cb.1.12480 (-);
ENAIOESO01	625501	630242	4742	2	2	3	3	2	3	2	2	3	2	2	2	2	2	2	2	2	2	2	2	2	3	3	2	2	3	3	2	3	
