# Supplementary material for: Genomic Variation among Strains of Crithidia bombi and C. expoeki
Source: mSphere. 2019 Sep 11;4(5):e00482-19. doi: 10.1128/mSphere.00482-19 (PMC6739494; doi:10.1128/mSphere.00482-19)
Supplement: TABLE S4 [file mSphere.00482-19-st004.pdf]

chrom	start	end	width	AK08_209	AK08_287	AK08_539	AK08_599	BJ08_064	BJ08_074	BJ08_083	BJ08_162	BJ08_163	BJ08_168	BJ08_172	BJ08_175	BJ08_193	Genes overlapping the region
ENA|OESE01(	1	6000	6000	1	1	1	1	2	2	2	2	4	2	2	2	2	
ENA|OESE01(	37801	38700	900	2	2	2	2	2	2	2	2	1	2	2	2	2	
ENA|OESE01(	3901	4800	900	2	2	2	2	2	2	2	1	2	2	2	2	2	
ENA|OESE01(	7201	8100	900	2	2	2	2	2	2	0	2	2	2	2	2	2	
ENA|OESE01(	119101	130085	10985	2	2	2	2	2	2	2	2	3	2	2	2	2	
ENA|OESE01(	46501	49200	2700	4	2	4	2	2	2	2	2	2	2	2	2	4	
ENA|OESE01(	51001	53400	2400	4	2	4	2	2	2	2	2	2	2	2	2	4	Ce.1.60510 (-);
ENA|OESE01(	129001	129900	900	0	2	0	2	2	2	2	2	2	2	2	2	2	
ENA|OESE01(	41101	42300	1200	2	2	2	2	2	2	2	2	1	2	2	2	2	
ENA|OESE01(	87901	89700	1800	2	2	2	2	2	2	2	2	2	4	2	2	2	
ENA|OESE01(	133201	134400	1200	2	2	2	2	2	2	2	2	2	2	2	2	2	Ce.1.03150 (-);
ENA|OESE01(	153901	157437	3537	1	1	1	1	2	2	2	2	2	2	2	2	2	
ENA|OESE01(	153901	154800	900	2	1	2	2	2	2	2	2	2	2	2	2	2	
ENA|OESE01(	1	11700	11700	2	2	2	1	2	2	2	2	2	2	1	2	2	
ENA|OESE01(	170401	171300	900	2	0	2	2	2	2	2	2	2	2	2	2	2	
ENA|OESE01(	1	1800	1800	2	2	2	2	1	2	2	2	2	2	2	2	2	
ENA|OESE01(	158401	159600	1200	2	2	2	2	2	2	2	2	2	2	2	1	2	
ENA|OESE01(	168301	169500	1200	2	2	2	0	2	2	2	2	2	2	2	2	2	
ENA|OESE01(	1	8100	8100	1	2	2	2	2	2	2	2	2	2	2	2	2	
ENA|OESE01(	112801	113700	900	2	2	2	2	2	2	2	2	1	2	2	2	2	
ENA|OESE01(	144601	146400	1800	4	2	4	3	2	2	2	2	2	2	2	2	2	
ENA|OESE01(	1	8100	8100	1	2	1	1	2	2	1	2	2	2	2	2	2	
ENA|OESE01(	8701	9900	1200	2	2	2	2	2	2	2	2	2	3	2	2	2	
ENA|OESE01(	35101	36000	900	2	2	2	2	2	2	2	2	1	2	2	2	1	
ENA|OESE01(	38401	40200	1800	2	2	2	2	2	2	2	2	3	2	2	2	2	
ENA|OESE01(	75601	76800	1200	2	2	2	2	2	2	2	2	2	2	2	2	1	
ENA|OESE01(	99601	102600	3000	2	2	2	2	2	2	2	2	1	2	2	2	2	
ENA|OESE01(	236101	237300	1200	2	2	2	2	2	2	2	2	2	2	4	2	2	Ce.1.34080 (+);
ENA|OESE01(	7501	8700	1200	2	2	3	3	2	1	2	2	2	1	2	2	2	Ce.1.75040 (+);
ENA|OESE01(	123601	127200	3600	3	2	2	2	2	2	2	2	2	2	2	2	2	Ce.1.74790 (+);
ENA|OESE01(	219601	240329	20729	1	2	2	2	2	2	2	2	2	2	2	2	2	Ce.1.74880 (+);
ENA|OESE01(	139201	143100	3900	2	2	2	2	2	2	2	2	1	2	2	2	2	
ENA|OESE01(	203101	204000	900	2	2	2	2	2	2	2	2	8	2	2	2	2	
ENA|OESE01(	228301	229200	900	2	2	2	2	2	2	2	2	1	2	2	2	2	
ENA|OESE01(	6601	7800	1200	2	0	0	0	2	2	2	2	2	2	2	2	2	
ENA|OESE01(	65101	70500	5400	2	3	3	4	2	1	6	2	1	1	2	1	1	
ENA|OESE01(	131701	146700	15000	2	2	2	2	2	2	1	2	2	2	2	2	2	
ENA|OESE01(	276901	287100	10200	2	2	2	2	2	2	2	2	3	2	2	2	4	Ce.1.32270 (-);
ENA|OESE01(	1	3000	3000	2	2	2	2	0	2	8	2	2	2	2	2	2	
ENA|OESE01(	300301	301967	1667	2	2	2	2	2	2	2	2	2	2	2	2	1	
ENA|OESE01(	11101	13200	2100	2	2	2	2	2	1	2	2	2	1	2	2	2	
ENA|OESE01(	273001	302100	29100	1	2	2	2	2	2	2	2	2	2	2	2	2	
ENA|OESE01(	1	2400	2400	2	2	2	2	1	2	2	2	2	2	2	2	2	
ENA|OESE01(	3901	5100	1200	0	0	0	2	2	2	2	2	2	2	2	2	2	
ENA|OESE01(	202801	204300	1500	2	2	2	2	2	2	2	2	0	2	2	2	1	
ENA|OESE01(	270601	271800	1200	2	2	2	2	2	2	2	2	1	2	2	2	2	
ENA|OESE01(	210001	211500	1500	2	2	2	2	3	2	2	2	2	2	2	2	2	
ENA|OESE01(	294001	295200	1200	2	2	2	2	2	2	2	2	1	2	2	2	2	
ENA|OESE01(	343501	344400	900	2	2	2	2	2	2	2	2	2	2	2	4	2	Ce.1.28870 (-);
ENA|OESE01(	1	4500	4500	1	1	1	1	2	2	2	2	2	2	2	2	2	
ENA|OESE01(	154501	155700	1200	2	2	2	2	2	2	2	2	1	2	2	2	1	
ENA|OESE01(	207901	234000	26100	1	2	5	2	1	3	0	2	1	1	2	2	3	
ENA|OESE01(	249301	251100	1800	2	2	5	2	2	2	2	2	2	3	2	2	2	
ENA|OESE01(	345901	347760	1860	1	1	1	1	2	2	2	2	2	2	2	2	2	
ENA|OESE01(	1	6600	6600	2	2	2	1	3	2	2	2	4	2	3	2	2	
ENA|OESE01(	261001	264000	3000	2	3	3	2	2	2	2	2	2	2	2	2	2	
ENA|OESE01(	359701	368400	8700	2	8	2	6	1	2	1	2	2	2	2	2	2	
ENA|OESE01(	4501	5700	1200	1	2	1	2	2	2	2	2	2	2	2	2	2	
ENA|OESE01(	151501	154200	2700	2	2	2	2	2	2	1	2	2	2	2	2	2	
ENA|OESE01(	173401	174300	900	2	2	2	4	2	2	2	2	2	2	2	2	2	
ENA|OESE01(	176101	182400	6300	2	2	2	2	2	2	2	2	2	2	3	2	2	
ENA|OESE01(	367501	371352	3852	2	2	8	2	2	2	1	2	2	2	2	1	2	
ENA|OESE01(	1	6000	6000	4	3	3	2	2	2	2	2	2	2	2	2	2	
ENA|OESE01(	170101	172800	2700	2	2	2	2	3	2	2	2	2	2	2	2	2	
ENA|OESE01(	362401	375681	13281	2	1	2	1	2	2	2	2	2	2	2	2	2	
ENA|OESE01(	4801	10800	6000	0	0	0	0	2	2	2	2	2	2	2	2	3	
ENA|OESE01(	263701	284700	21000	2	2	2	3	2	2	2	3	5	2	3	1	2	
ENA|OESE01(	403201	405589	2389	1	2	2	2	2	2	2	2	2	2	2	2	2	
ENA|OESE01(	19801	20700	900	1	2	2	2	2	2	2	2	2	2	2	2	2	Ce.1.56970 (-);
ENA|OESE01(	180001	181200	1200	2	2	2	2	2	2	2	2	1	2	2	2	2	
ENA|OESE01(	223801	231000	7200	1	2	3	2	2	2	2	2	2	2	2	2	3	
ENA|OESE01(	405901	407700	1800	1	1	1	1	2	2	2	2	2	2	2	2	2	Ce.1.57310 (-);
ENA|OESE01(	417601	418800	1200	2	2	2	2	2	2	2	2	4	2	2	2	2	Ce.1.57310 (-);
ENA|OESE01(	5401	7800	2400	1	1	1	0	2	2	2	2	2	2	2	2	2	
ENA|OESE01(	64201	69900	5700	2	2	2	2	2	2	2	2	4	2	2	2	2	
ENA|OESE01(	294901	296100	1200	2	2	2	2	2	2	2	2	4	2	2	2	2	Ce.1.43760 (+);
ENA|OESE01(	1	15300	15300	1	2	2	2	2	4	2	2	2	2	2	2	2	
ENA|OESE01(	407401	414900	7500	2	2	2	2	4	1	2	2	4	2	2	2	2	
ENA|OESE01(	460501	462363	1863	2	2	2	3	2	2	2	2	2	2	2	2	2	
ENA|OESE01(	123901	126000	2100	2	2	2	2	4	2	2	2	2	2	2	2	2	
ENA|OESE01(	139801	140700	900	1	2	2	2	2	2	2	2	2	2	2	2	2	
ENA|OESE01(	213601	216900	3300	4	2	2	2	2	2	2	2	2	2	2	2	2	
ENA|OESE01(	485701	489488	3788	2	1	1	0	2	2	2	2	2	2	2	2	2	
ENA|OESE01(	1	1500	1500	1	1	1	1	2	2	2	2	2	2	2	2	2	
ENA|OESE01(	194701	198000	3300	2	2	2	2	2	2	2	2	1	2	2	2	2	
ENA|OESE01(	292501	293400	900	2	2	2	2	0	2	2	2	2	2	2	2	2	
ENA|OESE01(	155701	158100	2400	2	2	2	2	2	1	2	2	2	2	2	2	2	
ENA|OESE01(	511201	512100	900	1	2	2	2	2	2	2	2	2	2	2	2	2	Ce.1.38270 (+);
ENA|OESE01(	102301	106200	3900	2	2	2	2	2	2	2	2	2	2	2	3	2	
ENA|OESE01(	204001	206400	2400	2	2	2	2	2	2	2	2	2	2	2	4	2	
ENA|OESE01(	289801	298800	9000	1	1	1	1	2	2	2	2	2	3	3	2	2	
ENA|OESE01(	369001	372600	3600	4	2	2	2	2	6	2	2	2	2	2	2	3	
ENA|OESE01(	378601	379500	900	2	2	2	2	2	2	2	2	1	2	2	2	2	
ENA|OESE01(	380701	386400	5700	3	2	4	3	2	2	2	2	2	2	2	2	2	
ENA|OESE01(	433801	434700	900	2	2	2	2	2	2	2	2	1	2	2	2	1	
ENA|OESE01(	481501	484200	2700	2	2	2	2	2	2	2	2	1	2	2	2	2	
ENA|OESE01(	495301	496200	900	2	2	2	4	2	2	2	2	2	2	2	2	2	Ce.1.71170 (-);
ENA|OESE01(	9001	12600	3600	2	2	2	2	2	2	2	2	2	3	2	2	2	
ENA|OESE01(	35101	36600	1500	2	2	2	2	2	2	2	2	1	2	2	2	1	
ENA|OESE01(	47101	48300	1200	2	2	2	2	2	2	2	2	2	2	3	2	2	
ENA|OESE01(	59701	60600	900	2	1	2	2	2	2	2	2	2	2	2	2	2	
ENA|OESE01(	202501	205200	2700	2	2	2	2	2	2	2	2	3	2	2	2	3	
ENA|OESE01(	344401	345300	900	2	2	2	2	2	2	2	2	2	2	2	2	1	
ENA|OESE01(	377401	378300	900	2	2	2	2	2	2	2	2	2	1	2	2	2	
ENA|OESE01(	387901	389400	1500	2	2	3	2	2	2	2	2	1	2	2	2	2	
ENA|OESE01(	545701	546600	900	2	2	2	2	2	2	2	2	5	2	2	2	2	
ENA|OESE01(	48901	59400	10500	1	2	2	2	2	2	2	2	2	2	2	2	2	
ENA|OESE01(	93001	96000	3000	2	2	2	2	3	2	2	2	2	2	2	2	2	
ENA|OESE01(	119401	138600	19200	2	2	2	2	2	1	2	2	2	1	4	2	2	
ENA|OESE01(	283501	287100	3600	2	2	2	2	2	2	2	2	2	1	2	2	2	Ce.1.05840 (+);
ENA|OESE01(	288301	292500	4200	2	2	2	2	2	2	2	2	2	2	2	2	1	
ENA|OESE01(	293701	299100	5400	2	2	4	2	2	2	2	2	2	2	2	2	1	
ENA|OESE01(	515401	516600	1200	2	2	1	2	2	2	2	2	2	2	2	2	2	
ENA|OESE01(	553201	560545	7345	1	1	1	1	2	2	2	2	2	2	2	2	2	
ENA|OESE01(	46801	47700	900	2	2	2	2	2	2	2	2	1	2	2	2	2	
ENA|OESE01(	53101	55500	2400	2	2	2	2	2	2	2	2	1	2	2	2	2	
ENA|OESE01(	154201	155100	900	2	2	2	2	2	2	2	3	2	2	2	2	2	
ENA|OESE01(	272101	279000	6900	2	0	2	1	2	2	2	4	2	2	2	2	2	
ENA|OESE01(	333301	334800	1500	2	2	2	2	2	2	2	2	3	2	2	2	2	
ENA|OESE01(	367201	368400	1200	2	2	2	2	2	2	2	2	2	2	2	1	2	
ENA|OESE01(	517501	518400	900	2	2	2	2	2	2	2	2	2	2	2	2	1	
ENA|OESE01(	10501	13200	2700	2	2	1	1	2	2	2	2	2	2	2	2	2	
ENA|OESE01(	215401	216600	1200	2	2	2	2	2	2	2	3	2	2	2	2	2	
ENA|OESE01(	287701	288900	1200	2	2	2	2	2	3	2	2	2	2	2	2	2	
ENA|OESE01(	305701	308400	2700	2	2	2	2	2	2	2	2	4	2	2	2	2	Ce.1.03720 (+);
ENA|OESE01(	317701	325800	8100	2	4	2	2	2	2	2	2	2	2	2	2	2	
ENA|OESE01(	401401	402300	900	2	2	2	2	2	2	2	2	2	2	2	2	1	
ENA|OESE01(	434701	435600	900	0	0	2	1	2	2	2	2	2	2	2	2	2	
ENA|OESE01(	440701	441600	900	3	3	2	3	2	2	2	2	2	2	2	2	2	
ENA|OESE01(	511801	512700	900	2	2	2	2	2	2	2	2	1	2	2	2	2	
ENA|OESE01(	3301	5100	1800	1	0	0	0	2	2	2	2	2	2	2	2	2	

ENA|OESE01(	36301	38400	2100	2	2	2	3	2	2	2	2	2	2	2	2	2	
ENA|OESE01(	180901	183000	2100	2	2	2	2	3	3	2	2	2	3	2	2	2	
ENA|OESE01(	216901	219000	2100	2	2	2	2	3	2	2	2	1	2	2	2	2	
ENA|OESE01(	235801	238500	2700	2	2	2	2	2	2	2	2	1	2	2	2	2	
ENA|OESE01(	405001	432000	27000	1	2	2	2	2	2	2	2	2	2	2	2	2	
ENA|OESE01(	491701	495900	4200	5	3	2	3	2	2	2	2	2	1	2	2	1	
ENA|OESE01(	563101	567600	4500	5	4	2	5	4	2	2	2	2	2	2	1	2	
ENA|OESE01(	161701	165300	3600	2	1	2	1	2	2	2	2	2	2	2	2	2	
ENA|OESE01(	127801	129900	2100	2	2	2	2	2	3	2	2	2	2	2	2	2	Ce.1.30350 (+);
ENA|OESE01(	306301	309300	3000	2	0	0	0	2	1	2	2	2	2	2	3	2	
ENA|OESE01(	322201	325500	3300	2	2	4	2	2	2	2	2	2	2	2	3	2	
ENA|OESE01(	430201	432300	2100	2	2	3	2	2	2	2	2	2	2	2	2	2	
ENA|OESE01(	1	1200	1200	2	7	7	7	2	2	2	2	2	2	2	2	2	
ENA|OESE01(	126901	128700	1800	2	2	2	2	2	4	2	2	2	2	2	2	2	
ENA|OESE01(	297001	297900	900	2	2	0	2	2	2	2	0	2	2	0	2	2	
ENA|OESE01(	328501	333000	4500	2	2	5	2	2	2	2	2	2	2	2	3	2	
ENA|OESE01(	479101	481500	2400	2	2	2	4	2	2	2	2	2	2	2	2	2	
ENA|OESE01(	482101	485700	3600	2	4	2	2	2	2	2	2	2	2	2	2	2	
ENA|OESE01(	593701	601800	8100	1	1	2	2	2	2	0	2	2	2	2	2	2	
ENA|OESE01(	1	12900	12900	1	1	1	1	2	2	2	2	2	6	2	2	2	
ENA|OESE01(	32101	33900	1800	2	2	2	2	2	2	2	2	1	2	2	2	2	
ENA|OESE01(	82801	83700	900	4	2	2	2	2	2	2	2	2	2	2	2	2	
ENA|OESE01(	453601	459900	6300	2	2	2	2	2	2	2	2	2	2	2	2	2	
ENA|OESE01(	467101	469200	2100	2	3	2	4	2	2	2	4	4	2	4	2	2	
ENA|OESE01(	607801	609124	1324	1	2	2	2	2	2	2	2	2	2	2	2	2	
ENA|OESE01(	510601	513900	3300	2	2	2	2	1	3	2	2	1	1	2	2	1	
ENA|OESE01(	546301	547500	1200	2	2	2	2	2	2	2	2	2	2	2	2	1	
ENA|OESE01(	686701	689400	2700	2	2	2	2	2	1	1	2	2	2	2	2	2	
ENA|OESE01(	210301	213300	3000	4	2	4	2	2	2	2	2	2	2	2	2	3	
ENA|OESE01(	233101	234900	1800	2	2	2	2	2	2	2	2	2	2	2	2	2	
ENA|OESE01(	297301	298200	900	2	2	2	2	2	2	2	2	2	1	2	2	2	
ENA|OESE01(	667801	669000	1200	4	2	2	2	2	2	2	2	2	1	2	2	2	
ENA|OESE01(	148801	151500	2700	2	2	1	2	2	2	2	2	2	2	2	2	2	Ce.1.50700 (-);
ENA|OESE01(	315001	316800	1800	2	2	2	2	2	2	2	2	2	2	2	2	1	
ENA|OESE01(	521701	524400	2700	1	2	2	2	2	2	2	2	2	2	2	2	2	
ENA|OESE01(	695401	714600	19200	2	2	2	2	2	2	2	2	2	4	2	2	2	
ENA|OESE01(	747601	753685	6085	1	1	2	2	2	2	2	2	2	2	2	2	2	
ENA|OESE01(	417601	418500	900	0	2	2	2	2	2	2	2	2	2	2	2	2	
ENA|OESE01(	453901	456600	2700	2	2	2	2	1	2	2	2	2	2	2	2	2	
ENA|OESE01(	645301	649200	3900	2	2	2	2	2	2	2	2	2	2	2	2	3	
ENA|OESE01(	1	3600	3600	2	1	2	2	2	2	2	2	2	2	2	4	2	Ce.1.41720 (+);
ENA|OESE01(	45901	48900	3000	2	1	2	3	6	2	2	1	1	1	1	1	1	
ENA|OESE01(	150901	182100	31200	1	1	1	1	2	1	2	2	4	1	2	2	1	
ENA|OESE01(	200701	204300	3600	2	2	2	2	2	2	2	2	1	2	1	1	1	
ENA|OESE01(	261901	267000	5100	2	2	2	2	2	2	2	3	3	2	3	2	5	
ENA|OESE01(	383101	384900	1800	1	2	2	2	2	2	2	2	2	2	2	2	2	
ENA|OESE01(	410401	413100	2700	2	2	3	2	2	2	2	2	2	2	2	2	2	
ENA|OESE01(	454501	455700	1200	2	2	2	2	2	2	2	2	2	2	4	2	2	
ENA|OESE01(	481201	482700	1500	2	2	2	6	2	2	2	6	2	2	6	2	2	
ENA|OESE01(	490201	491400	1200	2	2	2	6	2	2	2	2	2	2	6	2	2	Ce.1.42200 (-);
ENA|OESE01(	705601	707400	1800	2	2	2	2	2	2	2	2	1	2	2	2	1	
ENA|OESE01(	721201	735300	14100	2	2	2	2	2	2	2	2	3	2	2	2	2	Ce.1.42220 (-);
ENA|OESE01(	1	5400	5400	1	1	1	1	2	2	2	2	2	2	2	2	2	
ENA|OESE01(	74701	75900	1200	3	2	2	2	2	2	2	2	2	2	2	2	2	
ENA|OESE01(	364801	382200	17400	2	2	2	2	2	2	2	2	2	2	2	4	2	
ENA|OESE01(	872401	875400	3000	2	2	2	2	2	2	1	2	2	2	2	2	2	
ENA|OESE01(	6301	8100	1800	1	1	1	0	2	2	2	2	2	2	2	2	2	
ENA|OESE01(	27301	28200	900	2	2	2	2	2	2	1	2	2	2	2	2	2	
ENA|OESE01(	102301	107400	5100	2	1	2	2	2	2	2	2	2	2	2	2	2	
ENA|OESE01(	137101	138000	900	2	2	2	5	2	2	2	2	2	2	2	2	2	
ENA|OESE01(	378301	380700	2400	2	2	2	3	2	2	2	2	2	2	2	2	2	
ENA|OESE01(	422101	423300	1200	2	3	2	2	2	2	2	2	2	2	2	2	2	
ENA|OESE01(	675001	678600	3600	2	2	2	2	2	2	2	2	2	1	2	2	1	
ENA|OESE01(	684901	687300	2400	2	4	2	3	2	2	2	2	2	2	2	2	2	Ce.1.77420 (-);
ENA|OESE01(	717301	721500	4200	2	4	2	2	2	2	2	2	2	2	2	2	2	Ce.1.77280 (-);
ENA|OESE01(	873301	876900	3600	2	1	2	1	2	2	2	2	2	2	2	2	2	
ENA|OESE01(	883501	886285	2785	2	4	2	2	2	2	2	2	2	2	2	2	2	
ENA|OESE01(	1	900	900	2	2	8	2	2	2	2	2	2	2	2	2	2	
ENA|OESE01(	9901	23700	13800	1	2	2	2	2	2	2	2	2	2	2	2	2	
ENA|OESE01(	177001	177900	900	2	2	2	2	2	2	2	2	1	2	2	2	1	
ENA|OESE01(	243601	244500	900	2	2	2	2	2	2	2	2	1	2	2	2	2	
ENA|OESE01(	277201	278400	1200	1	2	1	2	2	2	2	2	2	2	2	2	2	Ce.1.53160 (+);
ENA|OESE01(	278701	280200	1500	2	2	2	2	2	2	2	2	3	2	2	2	2	Ce.1.52700 (+);
ENA|OESE01(	448801	453600	4800	2	2	2	2	2	2	2	2	1	2	2	2	1	
ENA|OESE01(	493501	496800	3300	2	2	2	2	2	2	3	2	2	2	2	2	3	Ce.1.51970 (+);
ENA|OESE01(	498301	499200	900	2	2	2	2	2	2	2	2	2	2	2	2	0	Ce.1.51820 (+);
ENA|OESE01(	603601	605100	1500	2	2	2	2	2	2	3	2	2	2	2	2	2	
ENA|OESE01(	612601	629700	17100	1	2	2	2	2	2	2	2	2	2	2	2	2	Ce.1.52990 (+);
ENA|OESE01(	772201	773400	1200	2	2	2	2	2	3	2	2	2	2	2	2	2	
ENA|OESE01(	831901	832800	900	2	2	2	2	2	2	2	2	2	2	2	2	1	
ENA|OESE01(	873601	906417	32817	2	2	2	2	2	4	2	2	2	2	2	2	2	
ENA|OESE01(	331501	333900	2400	2	2	2	2	2	2	1	2	2	2	2	2	2	
ENA|OESE01(	356101	361500	5400	2	3	2	2	2	2	1	3	2	2	4	2	2	
ENA|OESE01(	388201	398100	9900	2	2	2	2	2	2	2	2	4	2	2	2	2	
ENA|OESE01(	513901	517500	3600	2	2	2	2	2	2	2	2	1	2	2	2	2	
ENA|OESE01(	562801	581400	18600	1	1	1	2	2	2	2	2	2	2	2	2	2	
ENA|OESE01(	752101	753000	900	2	2	2	2	2	2	2	2	1	2	2	2	2	
ENA|OESE01(	878101	880800	2700	2	2	2	2	2	2	2	2	2	3	2	2	2	
ENA|OESE01(	1031401	1032300	900	2	1	2	2	2	2	2	2	2	2	2	2	2	
ENA|OESE01(	146101	151800	5700	2	2	2	2	2	2	2	2	2	2	2	2	2	
ENA|OESE01(	224401	225300	900	2	2	2	2	2	2	2	2	1	2	2	2	2	
ENA|OESE01(	309601	311100	1500	3	4	2	2	2	2	2	2	1	2	2	2	1	
ENA|OESE01(	345001	348300	3300	4	2	2	2	2	2	2	2	2	2	2	2	2	
ENA|OESE01(	666001	668400	2400	1	2	2	2	2	2	1	2	2	2	2	2	2	
ENA|OESE01(	756001	757200	1200	2	2	2	2	2	3	2	2	2	2	2	2	2	
ENA|OESE01(	828301	834000	5700	2	2	2	2	2	2	2	2	4	2	2	2	2	
ENA|OESE01(	1019401	1020600	1200	1	2	2	2	2	2	2	2	2	2	2	2	2	
ENA|OESE01(	1093501	1094400	900	2	2	2	2	2	2	2	2	3	2	2	2	2	Ce.1.09240 (+);
ENA|OESE01(	1151401	1152300	900	2	2	2	2	2	2	2	2	1	2	2	2	2	
ENA|OESE01(	1	13200	13200	1	1	0	2	2	2	2	2	2	4	2	2	4	
ENA|OESE01(	89401	91800	2400	2	2	2	2	2	3	2	2	2	3	2	2	2	
ENA|OESE01(	338401	339900	1500	2	2	2	2	2	2	2	2	1	2	2	2	2	
ENA|OESE01(	508201	509400	1200	2	2	2	2	2	2	2	2	1	2	2	2	2	
ENA|OESE01(	523501	525000	1500	2	2	2	2	2	2	3	2	2	2	2	2	2	
ENA|OESE01(	804001	804900	900	2	2	2	2	2	2	2	2	1	2	2	2	2	
ENA|OESE01(	1330801	1341300	10500	2	2	2	2	2	2	1	2	2	2	2	2	2	
ENA|OESE01(	1524301	1525800	1500	2	2	2	2	2	2	2	2	3	2	2	2	2	Ce.1.26160 (-);
ENA|OESE01(	1600201	1601100	900	2	2	2	2	2	2	2	2	1	2	2	2	2	
ENA|OESE01(	1669201	1670700	1500	2	2	2	2	2	2	2	2	2	2	3	2	2	
ENA|OESE01(	1921501	1926300	4800	0	2	1	0	2	2	2	2	3	2	2	1	2	
ENA|OESE01(	2196001	2197800	1800	2	2	2	2	2	2	0	2	2	2	1	2	2	
ENA|OESE01(	2333401	2336100	2700	2	2	2	3	2	2	2	2	2	2	2	2	2	
ENA|OESE01(	2514301	2515500	1200	2	2	2	2	2	2	2	2	1	2	2	2	1	
ENA|OESE01(	12001	12900	900	2	2	2	2	2	2	2	2	2	2	4	2	2	
ENA|OESE01(	357901	374700	16800	2	2	1	2	1	2	2	2	3	2	3	2	2	
ENA|OESE01(	491401	509700	18300	1	2	2	2	2	1	2	2	6	2	2	6	6	
ENA|OESE01(	541201	543000	1800	2	2	2	2	2	2	2	2	2	2	2	2	2	
ENA|OESE01(	585001	593100	8100	1	2	1	1	4	4	2	5	2	1	5	1	2	
ENA|OESE01(	746401	748500	2100	2	2	2	2	2	2	2	2	2	2	2	2	2	Ce.1.16650 (-);
ENA|OESE01(	750901	754500	3600	2	2	2	2	1	2	2	2	2	2	2	2	1	Ce.1.16650 (-);
ENA|OESE01(	1006801	1008300	1500	2	2	2	2	2	2	2	2	2	2	3	2	2	
ENA|OESE01(	1010701	1018500	7800	1	2	2	2	2	2	2	2	2	3	3	2	2	
ENA|OESE01(	1605901	1628700	22800	1	2	1	2	2	2	2	2	2	2	3	2	2	
ENA|OESE01(	1900501	1902900	2400	2	2	2	2	2	2	2	2	2	2	2	3	2	
ENA|OESE01(	2061601	2070000	8400	2	1	1	1	2	2	2	2	2	2	2	2	2	
ENA|OESE01(	2511001	2514600	3600	2	2	5	2	2	2	1	2	2	2	4	2	2	
ENA|OESE01(	2614801	2617200	2400	2	2	2	2	1	1	2	4	2	1	4	1	2	
ENA|OESE01(	3067501	3068400	900	2	1	2	2	2	2	2	2	2	2	2	2	2	
