# Supplementary material for: Genomic Variation among Strains of Crithidia bombi and C. expoeki
Source: mSphere. 2019 Sep 11;4(5):e00482-19. doi: 10.1128/mSphere.00482-19 (PMC6739494; doi:10.1128/mSphere.00482-19)
Supplement: TABLE S6 [file mSphere.00482-19-st006.pdf]

GO	Definition	Annotated	Significant	Expected	exactFisher, p-value	Species color code
A. Crithidia bombi, genes with positively selected sites						
**Biological process**						
1 GO:0007018	microtubule-based movement	92	21	8.73	8.6e-05	
2 GO:0006259	DNA metabolic process	176	29	16.70	0.0017	
3 GO:0006928	movement of cell or subcellular componen...	120	21	11.38	0.0036	
4 GO:0007017	microtubule-based process	138	23	13.09	0.0044	
5 GO:0015748	organophosphate ester transport	5	3	0.47	0.0073	
10 GO:0009190	cyclic nucleotide biosynthetic process	10	4	0.95	0.0105	
11 GO:0043412	macromolecule modification	463	57	43.93	0.0163	
12 GO:0009187	cyclic nucleotide metabolic process	12	4	1.14	0.0212	
14 GO:0006464	cellular protein modification process	412	50	39.09	0.0314	
15 GO:0036211	protein modification process	412	50	39.09	0.0314	
16 GO:0006869	lipid transport	8	3	0.76	0.0330	
17 GO:0030031	cell projection assembly	14	4	1.33	0.0369	
**Molecular function**						
1 GO:0005488	binding	2542	321	286.25	1.8e-05	
2 GO:0003774	motor activity	83	22	9.35	7.7e-05	
3 GO:0003777	microtubule motor activity	79	21	8.90	0.00011	
4 GO:0016462	pyrophosphatase activity	394	66	44.37	0.00033	
5 GO:0017111	nucleoside-triphosphatase activity	387	65	43.58	0.00034	
6 GO:0016818	hydrolase activity, acting on acid anhyd...	395	66	44.48	0.00035	
7 GO:0016817	hydrolase activity, acting on acid anhyd...	398	66	44.82	0.00044	
8 GO:0005524	ATP binding	633	93	71.28	0.00208	
9 GO:0032559	adenyl ribonucleotide binding	637	93	71.73	0.00256	
10 GO:0016887	ATPase activity	189	34	21.28	0.00317	
11 GO:0005515	protein binding	1053	142	118.58	0.00427	
12 GO:0030554	adenyl nucleotide binding	649	93	73.08	0.00459	
13 GO:0008144	drug binding	676	95	76.12	0.00746	
14 GO:0004012	phospholipid-translocating ATPase activi...	5	3	0.56	0.01191	
15 GO:0005548	phospholipid transporter activity	5	3	0.56	0.01191	
16 GO:0004608	phosphatidylethanolamine N-methyltransfe...	2	2	0.23	0.01265	
17 GO:0097367	carbohydrate derivative binding	748	101	84.23	0.01847	
18 GO:0004252	serine-type endopeptidase activity	10	4	1.13	0.01912	
19 GO:0016849	phosphorus-oxygen lyase activity	10	4	1.13	0.01912	
20 GO:0032553	ribonucleotide binding	743	100	83.67	0.02086	
21 GO:0005319	lipid transporter activity	6	3	0.68	0.02185	
22 GO:0035639	purine ribonucleoside triphosphate bindi...	727	97	81.87	0.02873	
23 GO:0032555	purine ribonucleotide binding	731	97	82.32	0.03295	
24 GO:0008017	microtubule binding	68	13	7.66	0.03716	
25 GO:0140097	catalytic activity, acting on DNA	76	14	8.56	0.04151	
B. Crithidia expoeki, genes with positively selected sites						
**Biological process**						
1 GO:0019219	regulation of nucleobase-containing comp...	96	10	3.94	0.0054	
2 GO:0006351	transcription, DNA-templated	103	10	4.23	0.0089	
3 GO:0097659	nucleic acid-templated transcription	103	10	4.23	0.0089	
4 GO:0032774	RNA biosynthetic process	104	10	4.27	0.0095	
5 GO:0051252	regulation of RNA metabolic process	90	9	3.69	0.0107	
6 GO:0055085	transmembrane transport	201	15	8.25	0.0165	
7 GO:0009187	cyclic nucleotide metabolic process	15	3	0.62	0.0214	
8 GO:0009190	cyclic nucleotide biosynthetic process	15	3	0.62	0.0214	
9 GO:0006355	regulation of transcription, DNA-templat...	54	6	2.22	0.0219	
10 GO:1903506	regulation of nucleic acid-templated tra...	54	6	2.22	0.0219	
11 GO:2001141	regulation of RNA biosynthetic process	54	6	2.22	0.0219	
12 GO:0033043	regulation of organelle organization	28	4	1.15	0.0259	
13 GO:0006188	IMP biosynthetic process	7	2	0.29	0.0307	
14 GO:0010638	positive regulation of organelle organiz...	7	2	0.29	0.0307	
15 GO:0016553	base conversion or substitution editing	7	2	0.29	0.0307	
16 GO:0016554	cytidine to uridine editing	7	2	0.29	0.0307	
**Molecular function**						
1 GO:0001882	nucleoside binding	131	12	6.01	0.016	
2 GO:0032549	ribonucleoside binding	131	12	6.01	0.016	
3 GO:0022804	active transmembrane transporter activit...	101	10	4.64	0.017	
4 GO:0016706	oxidoreductase activity, acting on paire...	14	3	0.64	0.024	
5 GO:0001883	purine nucleoside binding	126	11	5.79	0.029	
6 GO:0005525	GTP binding	126	11	5.79	0.029	
7 GO:0019001	guanyl nucleotide binding	126	11	5.79	0.029	

8	GO:0032550	purine ribonucleoside binding	126	11	5.79	0.029
9	GO:0032561	guanyl ribonucleotide binding	126	11	5.79	0.029
10	GO:0042626	ATPase activity, coupled to transmembran...	81	8	3.72	0.031
11	GO:0043492	ATPase activity, coupled to movement of ...	81	8	3.72	0.031
12	GO:0015399	primary active transmembrane transporter...	83	8	3.81	0.035
13	GO:0015405	P-P-bond-hydrolysis-driven transmembrane...	83	8	3.81	0.035
14	GO:0005488	binding	2931	146	134.57	0.036
15	GO:0046914	transition metal ion binding	347	23	15.93	0.044

C. Crithidia bombi, Switzerland strains

Biological process

1	GO:0055114	oxidation-reduction process	252	46	26.31	5.6e-05
2	GO:0030100	regulation of endocytosis	6	4	0.63	0.0015
3	GO:0006801	superoxide metabolic process	9	4	0.94	0.0096
4	GO:0060627	regulation of vesicle-mediated transport	9	4	0.94	0.0096

Molecular function

1	GO:0016491	oxidoreductase activity	306	52	30.25	3.7e-05
2	GO:0030234	enzyme regulator activity	37	11	3.66	0.00060
3	GO:0046906	tetrapyrrole binding	44	12	4.35	0.00081
4	GO:0004784	superoxide dismutase activity	6	4	0.59	0.00120
5	GO:0016721	oxidoreductase activity, acting on super...	6	4	0.59	0.00120
6	GO:0019904	protein domain specific binding	14	6	1.38	0.00135
7	GO:0020037	heme binding	41	11	4.05	0.00155
8	GO:0005198	structural molecule activity	153	27	15.12	0.00174
9	GO:0048037	cofactor binding	201	33	19.87	0.00197
10	GO:0098772	molecular function regulator	45	11	4.45	0.00347
11	GO:0019887	protein kinase regulator activity	12	5	1.19	0.00404
12	GO:0016616	oxidoreductase activity, acting on the C...	48	11	4.74	0.00590
13	GO:0019207	kinase regulator activity	13	5	1.29	0.00604
14	GO:0045309	protein phosphorylated amino acid bindin...	2	2	0.20	0.00975

D. Crithidia bombi, Alaskan strains

Biological process

1	GO:0044085	cellular component biogenesis	172	48	27.78	3.9e-05
2	GO:0044283	small molecule biosynthetic process	150	42	24.23	0.00011
3	GO:0032504	multicellular organism reproduction	29	13	4.68	0.00024
4	GO:0048609	multicellular organismal reproductive pr...	29	13	4.68	0.00024
5	GO:0009636	response to toxic substance	16	9	2.58	0.00027
6	GO:0044703	multi-organism reproductive process	30	13	4.85	0.00036
7	GO:0006913	nucleocytoplasmic transport	34	14	5.49	0.00041
8	GO:0051169	nuclear transport	34	14	5.49	0.00041
9	GO:0019953	sexual reproduction	28	12	4.52	0.00069
10	GO:0002164	larval development	32	13	5.17	0.00077

Molecular function

1	GO:0009922	fatty acid elongase activity	10	6	1.48	0.0013
2	GO:0003743	translation initiation factor activity	31	11	4.60	0.0035
3	GO:0016747	transferase activity, transferring acyl groups othe	74	20	10.97	0.0043
4	GO:0051537	2 iron, 2 sulfur cluster binding	10	5	1.48	0.0093

E. Crithidia bombi, all strains

Biological process

1	GO:0007018	microtubule-based movement	92	75	58.47	0.00011
2	GO:0006259	DNA metabolic process	176	134	111.85	0.00016
3	GO:0006281	DNA repair	99	79	62.92	0.00029
4	GO:0009100	glycoprotein metabolic process	23	22	14.62	0.00040
5	GO:0090304	nucleic acid metabolic process	455	320	289.17	0.00053
6	GO:0006486	protein glycosylation	22	21	13.98	0.00061
7	GO:0009101	glycoprotein biosynthetic process	22	21	13.98	0.00061
8	GO:0043413	macromolecule glycosylation	22	21	13.98	0.00061
9	GO:0070085	glycosylation	24	22	15.25	0.00192
10	GO:0006974	cellular response to DNA damage stimulus	117	89	74.36	0.00219
11	GO:0043412	macromolecule modification	463	321	294.25	0.00251
12	GO:0006464	cellular protein modification process	412	287	261.84	0.00284
13	GO:0036211	protein modification process	412	287	261.84	0.00284
14	GO:0006508	proteolysis	205	147	130.28	0.00653

Molecular function

1	GO:0030554	adenyl nucleotide binding	649	501	430.84	2.6e-11
2	GO:0032559	adenyl ribonucleotide binding	637	492	422.88	3.6e-11
3	GO:0005524	ATP binding	633	489	420.22	4.0e-11
4	GO:0008144	drug binding	676	516	448.77	3.3e-10
5	GO:0043168	anion binding	846	628	561.62	1.2e-08

6 GO:0097367	carbohydrate derivative binding	748	558	496.57	3.4e-08
7 GO:0032553	ribonucleotide binding	743	554	493.25	4.4e-08
8 GO:0032555	purine ribonucleotide binding	731	545	485.28	6.1e-08
9 GO:0017076	purine nucleotide binding	744	554	493.91	6.2e-08
10 GO:0035639	purine ribonucleoside triphosphate bindi...	727	542	482.63	6.8e-08
11 GO:0005488	binding	2542	1755	1687.53	9.1e-08
12 GO:0016787	hydrolase activity	895	651	594.15	1.6e-06
13 GO:0036094	small molecule binding	962	696	638.63	2.2e-06
14 GO:0000166	nucleotide binding	913	661	606.10	4.0e-06
15 GO:1901265	nucleoside phosphate binding	913	661	606.10	4.0e-06
16 GO:0017111	nucleoside-triphosphatase activity	387	295	256.91	5.7e-06
17 GO:0016462	pyrophosphatase activity	394	299	261.56	9.5e-06
18 GO:0016818	hydrolase activity, acting on acid anhyd...	395	299	262.22	1.4e-05
19 GO:0016817	hydrolase activity, acting on acid anhyd...	398	301	264.22	1.5e-05
20 GO:0003774	motor activity	83	72	55.10	1.8e-05
21 GO:0043167	ion binding	1320	932	876.29	2.3e-05
22 GO:0003777	microtubule motor activity	79	68	52.44	5.5e-05
23 GO:0005515	protein binding	1053	746	699.04	0.00014
24 GO:0008017	microtubule binding	68	57	45.14	0.00097
25 GO:0004386	helicase activity	97	78	64.39	0.00151
26 GO:0015631	tubulin binding	70	58	46.47	0.00157
27 GO:0016887	ATPase activity	189	144	125.47	0.00174
28 GO:0008237	metallopeptidase activity	47	40	31.20	0.00323
29 GO:0004806	triglyceride lipase activity	13	13	8.63	0.00481
30 GO:0008236	serine-type peptidase activity	18	17	11.95	0.00625
31 GO:0017171	serine hydrolase activity	18	17	11.95	0.00625
32 GO:0003690	double-stranded DNA binding	12	12	7.97	0.00726

F. Crithidia expoeki, Switzerland strains

**Biological process**					
1 GO:0042180	cellular ketone metabolic process	19	8	1.48	4.4e-05
2 GO:0006744	ubiquinone biosynthetic process	15	7	1.17	6.2e-05
3 GO:0042181	ketone biosynthetic process	15	7	1.17	6.2e-05
4 GO:1901663	quinone biosynthetic process	15	7	1.17	6.2e-05
5 GO:0006120	mitochondrial electron transport, NADH t...	11	6	0.86	7.1e-05
6 GO:0006743	ubiquinone metabolic process	16	7	1.25	0.00010
7 GO:1901661	quinone metabolic process	16	7	1.25	0.00010
8 GO:0006814	sodium ion transport	13	6	1.02	0.00023
9 GO:0018205	peptidyl-lysine modification	25	8	1.95	0.00042
10 GO:0022904	respiratory electron transport chain	28	7	2.19	0.00467
11 GO:0042773	ATP synthesis coupled electron transport	28	7	2.19	0.00467
12 GO:0042775	mitochondrial ATP synthesis coupled elec...	28	7	2.19	0.00467
13 GO:0022900	electron transport chain	29	7	2.27	0.00576
14 GO:0002182	cytoplasmic translational elongation	6	3	0.47	0.00790
15 GO:0008612	peptidyl-lysine modification to peptidyl...	6	3	0.47	0.00790
16 GO:1900247	regulation of cytoplasmic translational ...	6	3	0.47	0.00790
17 GO:2000765	regulation of cytoplasmic translation	6	3	0.47	0.00790
18 GO:0030001	metal ion transport	46	9	3.59	0.00800
19 GO:0044237	cellular metabolic process	2147	187	167.78	0.00825
**Molecular function**					
1 GO:0003954	NADH dehydrogenase activity	12	7	0.94	9.4e-06
2 GO:0008137	NADH dehydrogenase (ubiquinone) activity	11	6	0.86	7.2e-05
3 GO:0050136	NADH dehydrogenase (quinone) activity	11	6	0.86	7.2e-05
4 GO:0016811	hydrolase activity, acting on carbon-nit...	31	9	2.42	0.00042
5 GO:0016651	oxidoreductase activity, acting on NAD(P...	38	9	2.97	0.00207
6 GO:0016655	oxidoreductase activity, acting on NAD(P...	19	6	1.48	0.00246
7 GO:0051539	4 iron, 4 sulfur cluster binding	9	4	0.70	0.00336
8 GO:0016810	hydrolase activity, acting on carbon-nit...	56	11	4.38	0.00341
9 GO:0016209	antioxidant activity	22	6	1.72	0.00553
10 GO:0051536	iron-sulfur cluster binding	36	8	2.81	0.00554
11 GO:0051540	metal cluster binding	36	8	2.81	0.00554
12 GO:0004743	pyruvate kinase activity	2	2	0.16	0.00609
13 GO:0030955	potassium ion binding	2	2	0.16	0.00609
14 GO:0031420	alkali metal ion binding	2	2	0.16	0.00609
15 GO:0042586	peptide deformylase activity	2	2	0.16	0.00609

I. Crithidia expoeki, Alaskan strains

**Biological process**					
1 GO:0017004	cytochrome complex assembly	8	7	2.30	0.00095
2 GO:0043412	macromolecule modification	533	179	152.92	0.00431
3 GO:0098771	inorganic ion homeostasis	16	10	4.59	0.00493

4	GO:0006810	transport	566	188	162.39	0.00590
5	GO:0051234	establishment of localization	570	189	163.54	0.00627
6	GO:0055085	transmembrane transport	201	74	57.67	0.00632
7	GO:0051179	localization	624	205	179.03	0.00696
8	GO:0009069	serine family amino acid metabolic proce...	194	71	55.66	0.00868
9	GO:0033108	mitochondrial respiratory chain complex ...	6	5	1.72	0.00882
10	GO:0006468	protein phosphorylation	204	74	58.53	0.00950
**Molecular function**						
1	GO:0005515	protein binding	1005	360	308.51	3.6e-05
2	GO:0016667	oxidoreductase activity, acting on a sul...	35	20	10.74	0.0010
3	GO:0003747	translation release factor activity	5	5	1.53	0.0027
4	GO:0008079	translation termination factor activity	5	5	1.53	0.0027
5	GO:0015036	disulfide oxidoreductase activity	27	15	8.29	0.0061
6	GO:0005388	calcium-transporting ATPase activity	4	4	1.23	0.0089
7	GO:0015085	calcium ion transmembrane transporter ac...	4	4	1.23	0.0089

J. Crithidia expoeki, all strains

**Biological process**						
1	GO:0043412	macromolecule modification	533	157	116.48	4.9e-06
2	GO:0007018	microtubule-based movement	97	38	21.20	6.8e-05
3	GO:0006974	cellular response to DNA damage stimulus	88	35	19.23	9.2e-05
4	GO:0006281	DNA repair	82	33	17.92	0.00011
5	GO:0006259	DNA metabolic process	172	58	37.59	0.00016
6	GO:0090304	nucleic acid metabolic process	534	149	116.70	0.00021
7	GO:0006928	movement of cell or subcellular componen...	131	46	28.63	0.00027
8	GO:0070647	protein modification by small protein co...	54	23	11.80	0.00046
9	GO:0009451	RNA modification	75	29	16.39	0.00063
10	GO:0006464	cellular protein modification process	459	128	100.31	0.00065
11	GO:0036211	protein modification process	459	128	100.31	0.00065
12	GO:0016579	protein deubiquitination	19	11	4.15	0.00067
13	GO:0070646	protein modification by small protein re...	19	11	4.15	0.00067
14	GO:0009069	serine family amino acid metabolic proce...	194	61	42.40	0.00091
15	GO:0000723	telomere maintenance	12	8	2.62	0.00107
16	GO:0032200	telomere organization	12	8	2.62	0.00107
17	GO:0010038	response to metal ion	8	6	1.75	0.00199
18	GO:0006468	protein phosphorylation	204	62	44.58	0.00213
19	GO:0033554	cellular response to stress	109	37	23.82	0.00214
20	GO:0032011	ARF protein signal transduction	4	4	0.87	0.00227
21	GO:0032012	regulation of ARF protein signal transdu...	4	4	0.87	0.00227
22	GO:0046578	regulation of Ras protein signal transdu...	4	4	0.87	0.00227
23	GO:0051056	regulation of small GTPase mediated sign...	4	4	0.87	0.00227
24	GO:0043170	macromolecule metabolic process	1442	350	315.12	0.00237
25	GO:0046834	lipid phosphorylation	6	5	1.31	0.00242
26	GO:0046854	phosphatidylinositol phosphorylation	6	5	1.31	0.00242
27	GO:0030258	lipid modification	14	8	3.06	0.00425
28	GO:0060249	anatomical structure homeostasis	14	8	3.06	0.00425
29	GO:0001522	pseudouridine synthesis	26	12	5.68	0.00493
30	GO:0006260	DNA replication	67	24	14.64	0.00585
31	GO:0009187	cyclic nucleotide metabolic process	15	8	3.28	0.00738
32	GO:0009190	cyclic nucleotide biosynthetic process	15	8	3.28	0.00738
33	GO:0007017	microtubule-based process	163	49	35.62	0.00764
34	GO:0006644	phospholipid metabolic process	73	25	15.95	0.00949
**Molecular function**						
1	GO:0008144	drug binding	756	242	181.00	1.3e-08
2	GO:0030554	adenyl nucleotide binding	730	234	174.78	2.2e-08
3	GO:0032559	adenyl ribonucleotide binding	718	230	171.91	3.3e-08
4	GO:0005524	ATP binding	717	229	171.67	4.8e-08
5	GO:0016817	hydrolase activity, acting on acid anhyd...	468	156	112.05	6.4e-07
6	GO:0017111	nucleoside-triphosphatase activity	455	152	108.94	7.9e-07
7	GO:0016462	pyrophosphatase activity	462	153	110.61	1.3e-06
8	GO:0004386	helicase activity	102	46	24.42	1.7e-06
9	GO:0016887	ATPase activity	237	88	56.74	2.0e-06
10	GO:0016818	hydrolase activity, acting on acid anhyd...	465	153	111.33	2.0e-06
11	GO:0017076	purine nucleotide binding	852	255	203.99	3.9e-06
12	GO:0032555	purine ribonucleotide binding	839	251	200.88	5.1e-06
13	GO:0035639	purine ribonucleoside triphosphate bindi...	838	250	200.64	6.8e-06
14	GO:0016787	hydrolase activity	1028	297	246.13	1.4e-05
15	GO:0008026	ATP-dependent helicase activity	74	34	17.72	2.4e-05
16	GO:0070035	purine NTP-dependent helicase activity	74	34	17.72	2.4e-05
17	GO:0097367	carbohydrate derivative binding	901	260	215.72	7.0e-05
18	GO:0032553	ribonucleotide binding	897	257	214.76	0.00014

19	GO:0003777	microtubule motor activity	83	35	19.87	0.00016
20	GO:0140097	catalytic activity, acting on DNA	70	30	16.76	0.00034
21	GO:0005488	binding	2931	744	701.75	0.00039
22	GO:0005515	protein binding	1005	279	240.62	0.00073
23	GO:0003774	motor activity	89	35	21.31	0.00080
24	GO:0051020	GTPase binding	11	8	2.63	0.00086
25	GO:0043168	anion binding	1032	282	247.08	0.00205
26	GO:0003678	DNA helicase activity	25	13	5.99	0.00216
27	GO:0042623	ATPase activity, coupled	192	63	45.97	0.00271
28	GO:0005086	ARF guanyl-nucleotide exchange factor ac...	4	4	0.96	0.00327
29	GO:0004672	protein kinase activity	190	62	45.49	0.00338
30	GO:0004518	nuclease activity	84	31	20.11	0.00495
31	GO:0000166	nucleotide binding	1107	297	265.04	0.00510
32	GO:1901265	nucleoside phosphate binding	1107	297	265.04	0.00510
33	GO:0004527	exonuclease activity	36	16	8.62	0.00533
34	GO:0140098	catalytic activity, acting on RNA	126	43	30.17	0.00567
35	GO:0016773	phosphotransferase activity, alcohol gro...	249	77	59.62	0.00573
36	GO:0016866	intramolecular transferase activity	31	14	7.42	0.00756
37	GO:0016849	phosphorus-oxygen lyase activity	14	8	3.35	0.00773
38	GO:0036094	small molecule binding	1183	313	283.24	0.00954
